# Healthy adults supplemented with a nutraceutical formulation containing *Aloe vera* gel, rosemary and *Poria cocos* enhances the effect of influenza vaccination in a randomized, triple-blind, placebo-controlled trial

**DOI:** 10.3389/fnut.2023.1116634

**Published:** 2023-04-24

**Authors:** Erin D. Lewis, David C. Crowley, Najla Guthrie, Malkanthi Evans

**Affiliations:** KGK Science Inc., London, ON, Canada

**Keywords:** nutraceutical, immunity, vaccination, *Aloe vera* gel, rosemary, *Poria cocos*

## Abstract

The study objective was to examine the role of a formulation, UP360, containing rosemary and *Poria cocos* extracts and *Aloe vera* gel powder, in healthy adults on supporting immune function with influenza vaccination. A 56-day randomized, triple-blind, placebo-controlled, parallel study consisted of a 28-day pre-vaccination period, an influenza vaccination on Day 28 and a 28-day post-vaccination period. Men and women ages 40–80 who had not yet been vaccinated for the flu were randomized to UP360 or Placebo (*n* = 25/group). At baseline, Days 28 and 56, blood lymphocyte populations, immunoglobulins (Ig), and cytokines were measured, and quality of life (QoL) questionnaires administered. The Wisconsin Upper Respiratory Symptom Survey (WURSS)-24 was completed daily by participants to measure incidence of upper respiratory tract infection (URTIs). In the post-vaccination period, TCR gamma-delta (γδ+) cells, known as γδ T cells, increased with UP360 supplementation compared to Placebo (*p* < 0.001). The UP360 group had a 15.6% increase in influenza B-specific IgG levels in the post-vaccination period (*p* = 0.0006). UP360 significantly increased the amount of circulating glutathione peroxidase (GSH-Px) from baseline at Day 28 (*p* = 0.0214), an enzyme that is important for neutralizing free radicals. While UP360 supplementation initially decreased levels of anti-inflammatory cytokine IL-1RA in the pre-vaccination period, IL-1RA levels were increased in the post-vaccination period (*p* ≤ 0.0482). Levels of IL-7 increased from baseline at Day 56 with UP360 supplementation (*p* = 0.0458). Despite these changes in immune markers, there were no differences in URTI symptoms or QoL between UP360 and Placebo. These results suggest UP360 supplementation was beneficial in eliciting a healthy, robust immune response in the context of vaccination. No changes in subjective measures of URTI illness or QoL demonstrated that participants’ QoL was not negatively impacted by UP360 supplementation. There were no differences in clinical chemistry, vitals or adverse events confirming the good safety profile of UP360. The trial was registered on the International Clinical Trials Registry Platform (ISRCTN15838713).

## 1. Introduction

Globally, one of the leading causes of morbidity and mortality are acute respiratory tract infections, illustrated by seasonal flu epidemics and most recently COVID-19, caused by the SARS-CoV2 infection. In Canada, seasonal influenza is the leading cause of death due to the vaccine-preventable disease (1) with 3,500 mortalities per year (2). In the United States and Canada, the flu season occurs in the fall and winter, peaking anywhere from late November through March (2). During these months, influenza increases the demand on the healthcare system with 12,000 hospitalizations occurring annually (3). The prevalence of the flu during this period also translates into billions of dollars in lost revenue and worker productivity with over 45 million days of work lost annually, translating into an estimated $40 billion USD lost to both direct and indirect costs (4).

Presently, there is far greater strain on the health care system due to the COVID-19 pandemic as co-infection with COVID-19 and influenza or other respiratory pathogens is a significant risk factor for prolonged hospitalization (5, 6). Amid this public health crisis, it is critical to minimize the burden of influenza on the healthcare system so resources can be allocated appropriately. Further, a recent study suggested influenza vaccination in an older population over the age of 65 was negatively associated with COVID-19 mortality (7), supporting the importance of vaccination during this time. These significant public health implications point to the urgent need to increase vaccine adoption. However the low adoption rates of vaccination (8), combined with low effectiveness (9, 10) highlight the need to find therapies that may increase the efficacy of the flu vaccine to prevent illness, while reducing rates of upper respiratory tract infections (URTIs).

The role of nutraceuticals on supporting immune function and response with influenza (11–14) and COVID-19 (15) vaccination has been explored. A formulation containing extracts of *Acacia catechu (Senegalia catechu)* and *Scutellaria baicalensis* increased glutathione peroxidase (GSH-Px) prior to influenza vaccination and increased total IgA and influenza B-specific IgG levels following vaccination in healthy adults (16) and suggested benefit in mounting a robust humoral response after vaccination.

Using the same model, the current study investigated a nutraceutical formulation, UP360, containing rosemary and *Poria cocos* extracts and *Aloe vera* gel powder on modulation of immune responses after administering a vaccine in a healthy adult population. *Aloe vera* leaf gel contains a variety of bioactive ingredients including vitamins, minerals, anthraquinones, and polysaccharides, that have antioxidant, anti-inflammatory, and anti-bacterial effects (17–20). *In vitro* studies have shown that aloe polysaccharides inhibits the replication of the H1N1 influenza virus (17) and animal models demonstrated reductions in viral load and clinical symptoms of influenza infection and increased survival and Ig production following influenza immunization (17, 21). Several components of rosemary leaf have immunomodulatory effects *in vitro* and in animal models (22), primarily through increasing the IgM and IgG response following immune challenges (23). *Poria cocos* is a medicinal mushroom also known as fuling, matsuhodo, poria or China root. The major bioactive is the polysaccharide β-glucan that has been shown to have anti-inflammatory and immunomodulatory effects (24), with activation of Natural Killer (NK) cells a potential mechanism of action. The adjuvant activity of *Poria cocos* has been examined in a variety of animal models with rabies, H1N1 influenza and hepatitis B vaccines (25, 26). Therefore, the objective of the current study was to examine the role of UP360 on immune function support with influenza vaccination in healthy adults.

## 2. Materials and methods

### 2.1. Study design and ethics approvals

This randomized, triple-blind, placebo-controlled, parallel clinical trial was conducted at KGK Science Inc. (London, Ontario, Canada) between February 17 to May 21, 2021. The complete study design has been reported previously and the current publication will report on UP360 compared to Placebo only. UP360 has unique immunomodulatory effects and mechanism of action compared to UP446 (16).

This study was approved by the Natural and Non-Prescription Health Products Directorate (NNHPD), Health Canada, Ottawa, Ontario on February 1, 2021. Approval from the Research ethics board was granted from the Institutional Review Board (IRB) Services, Aurora, Ontario on February 2, 2021. The study was conducted according to the International Council for Harmonization of Technical Requirements for Pharmaceuticals for Human Use (ICH) Guideline for Good Clinical Practice (GCP) and in compliance with the Declaration of Helsinki guidelines and subsequent amendments. The study followed the CONSORT guidelines for randomized controlled trials (27) (Supplementary Table 1) and the full clinical trial was registered on the International Clinical Trials Registry Platform with the registration number ISRCTN15838713. Prior to any procedures being initiated, written informed consent was obtained from all study participants.

### 2.2. Study participants

Individuals were required to meet the following inclusion criteria: male or female, 40 to 80 years of age; not received their influenza vaccine for the 2020/2021 season but willing to receive it during the study; agreed to provide verbal history of flu vaccination, agreed to complete study questionnaire and associated diaries, and attend and complete clinic visits; agreed to maintain their current lifestyle habits including diet, sleep, and exercise, as well maintain their existing supplement and medication routine.

Individuals were excluded if they had an allergy to the vaccine or investigational products (IPs); were unvaccinated and contracted the flu prior to baseline or study vaccination; had a COVID-19 diagnosis prior to baseline or study vaccination; were vaccinated for COVID-19; used herbal medicines or supplements to modulate the immune system unless willing to washout for specified periods prior to enrolment; unable to give informed consent and/or were cognitively impaired; or had any other condition, lifestyle factor or chronic disease that in the Medical Director’s (MD) opinion, may have adversely affected the ability of the participant to complete the study or its assessments, or posed a significant risk to the participant (Supplementary Table 2).

### 2.3. Investigational products and vaccine

UP360, contained a formulation of rosemary and *Poria cocos* extracts and *Aloe vera* gel powder. Briefly, fresh *Aloe vera* leaves were washed, and the outer rind removed. The whole leaf gel was treated with cellulose enzymes and filtered through activated charcoal. Using Qmatrix® processing, filtrates were concentrated using low pressure evaporation and dehydrated to a dry powder. The *Aloe vera* leaf gel powder was produced as a lyophilizate with an extraction ratio of 200:1 containing no less than 10% polysaccharides. The molecular weight distribution of Aloe polysaccharide was between 50 and 200 kDalton (kDa) with average molecular weight at 80 kDa. The rosemary leaf extract was produced by ethanol/water extraction containing no less than 30% rosmarinic acid with an extraction ratio of 100:1. Ground *Poria cocos* or *Wolfiporia extensa* scleroticum powder was extracted with ethanol and water to give the ethanol precipitate with no less than 20% polysaccharides at an extraction ratio of 15–18:1. *Poria cocos* scleroticum extract*, Aloe vera* leaf gel powder, and rosemary leaf extract were blended at a ratio of 6:3:1 by weight to provide the final composition of UP360. Standardized UP360 contained no less than 10% total polysaccharides from both *Aloe gel* powder and *Poria cocos* extract, and not less than 2% rosmarinic acid from rosemary leaf extract.

Excipients included polydextrose, magnesium stearate, and microcrystalline cellulose (MCC). The placebo also contained excipients magnesium stearate and MCC. Both UP360 and placebo were manufactured by Acenzia (Tecumseh, Ontario, Canada).

Participants were instructed to take their respective study product twice daily, once in the morning and once in the evening around mealtimes with food approximately 4–6 ounces of water for 56 days.

At Day 28, participants were given an intramuscular injection of the 2020/2021 specific influenza vaccine (FLUCELVAX® QUAD, Seqirus, Kirkland, QC). The vaccine contained strains for Haemagglutinin A/Hong Kong/45/2019 (H3N2)-like virus (A/Delaware/39/2019), Haemagglutinin A/Hawaii/70/2019 (H1N1) pdm09-like virus (A/Nebraska/14/2019), Haemagglutinin B/Phuket/3073/2013-like virus (B/Singapore/INFTT-16-0610/2016) and Haemagglutinin B/Washington/02/2019-like virus (B/Darwin/7/2019).

### 2.4. Randomization and blinding

At baseline, a blinded investigator assigned a randomization number to all eligible participants based on the study randomization list.^1^ The investigator was provided a randomization schedule indicating the randomization order. Investigators, all study personnel including the statistician, and participants were blinded to the study products.

The Placebo and UP360 supplement contained similar excipients and matched in their appearance to ensure allocation concealment. The study products were packaged in sealed bottles identical in appearance and labelled according to ICH GCP and applicable local regulatory guidelines. The study products were labelled by unblinded personnel not involved in the conduct of the study.

### 2.5. Outcome measures

The efficacy of UP360 on supporting immune function was assessed by immune parameters including blood lymphocyte populations (CD3+, CD4+, CD8+, TCRγδ+, CD3-CD16 + 56+ and CD45+), immunoglobulins, influenza-specific antibodies, serum, cytokines, and glutathione peroxidase (GSH-Px). The severity, incidence, frequency of URTI symptoms, flu and COVID-19 cases, and hospitalizations, quality of life and over-the-counter cold and flu medication use were assessed.

Safety outcomes included vital signs, clinical chemistry, and post-emergent adverse events. Clinical chemistry parameters including liver function (alanine aminotransferase (ALT), alkaline phosphatase (ALP), aspartate aminotransferase (AST), and total bilirubin), kidney function (estimated glomerular filtration rate (eGFR), creatinine, sodium, potassium and chloride), and glucose were analyzed from blood drawn at each study visit by Dynacare (London, ON, Canada) using standard laboratory procedures.

### 2.6. Study procedures

At the screening, eligibility was assessed *via* medical history and current health status, inclusion and exclusion criteria, concomitant therapies, vaccination history, clinical chemistry, and hematology. Three days prior to baseline, eligible participants completed a daily diary including the Wisconsin Upper Respiratory Symptom Survey (WURSS)-24 to capture the frequency, incidence, and severity of URTI symptoms (28). Responses on the WURSS-24 were also used to identify participants reporting COVID-19 or flu as assessed by the MD. Participants determined to have COVID-19 or flu prior to baseline were removed from the study.

At baseline (Day 0), Days 28, and 56, blood was collected to assess lymphocyte populations, immunoglobulins, cytokines, and clinical chemistry, described in detail below. Blood pressure and heart rate were measured and a vitality and quality of life (QoL) questionnaire and COVID-19-Impact on Quality of Life (COV19-QoL) scale v1.5 (29) was administered at each study visit. TheCOV19-QoL was used to evaluate the effect of the COVID-19 pandemic on the wellbeing of participants.

Daily study diaries were completed by participants to capture rates and hospitalizations of influenza and COVID-19 infections, use of over-the-counter cold and flu medications, and changes in health. The WURSS-24 was part of the daily diary completed throughout the 56-day study period. At each study visit study diaries were reviewed to ensure the wellbeing and safety of participants and outcomes related to influenza or COVID-19 infection were evaluated. A urine pregnancy test was conducted for women of childbearing potential at baseline and Day 56 at the KGK clinic.

At the Day 28 visit, participants received an influenza vaccination, as described above. The “pre-vaccination” period consisted of baseline to Day 28 and “post-vaccination” was Days 28 to 56. Participants consumed the investigational products (IPs) daily for a total of 56 days.

As a measure of safety, adverse events (AEs) were assessed throughout the study period using study diaries. The classification of AEs was made based on the description, frequency, duration, intensity, and outcome and the MD assessed each AE for causality and intensity. The Medical Dictionary for Regulatory Activities (MEDRA) terminology (version 22.0) was used for AE coding.

### 2.7. Laboratory analyses

Flow cytometry was used to analyze lymphocyte populations (London Health Sciences Centre Laboratory, London, ON). Briefly, a whole blood sample was incubated with fluorescent-tagged monoclonal antibodies that were specific for the populations of interest (T and B lymphocytes and NK cells). A 10-colour Navios instrument was used, and the output was analyzed using Kaluza software (Beckman Coulter, Brea, CA, USA). Analysis of two and four-colour tubes with antigen gating and CD45 were used to examine the lymphocyte populations and absolute counts were determined using a bead-based method, known as single platform testing.

Analysis of immunoglobulins was completed using Roche Cobas c701 analyzer (LifeLabs, London, ON). The adult reference ranges were 0.5–4.17 g/L for IgA, 6–16 g/L for IgG and 0.3–2.30 g/L for IgM. Influenza-specific antibodies were analyzed using Qualitative ELISAs for Human Influenza A IgA ELISA (Abcam # ab108743), Human Influenza A IgG ELISA (Abcam # ab108745), Human Influenza A IgM ELISA (Abcam # ab108747), Human Influenza B IgA ELISA (Abcam # ab108744), Human Influenza B IgG ELISA (Abcam # ab108746), Human Influenza B IgM ELISA (Abcam # ab108748). For influenza A-specific antibodies, the observed reference ranges were 0–28, 0–73 and 0–15 for IgA, IgG and IgM, respectively. The observed reference ranges for influenza B-specific IgA, IgG and IgM were 0–18, 0–29 and 0–26, respectively.

Serum cytokines were analyzed by Sirona Dx (Portland, OR, USA). Samples were thawed on ice and centrifuged at 10,000 x g for 10 min at 4°C after which the serum was transferred to a new tube and placed on ice until being tested. Serum was tested using the Luminex Cytokine 25-Plex Human ProcartaPlex Panel 1B ImmunoAssay, Luminex xMAP Technology. The samples were analyzed using a Magpix instrument (Thermofisher). The analytical ranges were as follows: 31–342,641 pg./ml for IL-1RA, 0.46–1927 for IL-1α, 0.84–5,876 pg./ml for IL-7, and 4.9–52,126 for IL-21. The concentration of GSH-Px was measured using a quantitative colorimetric method (Glutathione Peroxidase Assay Kit (Abcam # ab102530)) with an analytical range of 0–333 mU/ml. The amount of GSH-Px enzyme produced is proportional to the amount of glutathione disulfide (GSSG) from reduced glutathione (GSH) which was measured by the amount of NADPH consumed through the reduction of GSSG to GSH.

### 2.8. Compliance

To assess compliance, participants were asked bring all unused and open packages to each study visit. The percent of compliance was calculated by dividing the number of dosage units actually taken by the number of dosage units expected to be taken, then multiplied by 100. If there was a discrepancy between the amount of study product returned and the compliance information recorded in the study diary, compliance was calculated based on the product returned unless the participant provided an explanation for the loss.

### 2.9. Statistical analyses

A planned total sample size of 75 participants, with 25 participants randomized to each study group (30–32) was calculated using a sample size calculation previously described by Lewis et al. (16).

The analysis of study endpoints were done as continuous variables and descriptive statistics including number of participants, mean, standard deviation, median, minimum, and maximum values were presented for each visit. Descriptive statistics were also reported for the changes from baseline (Day 0) to Days 28 (pre-vaccination), Day 28 to Day 56 (post-vaccination), and baseline to Day 56 (end of study). Endpoints collected from study diaries in the between-visit intervals were reported as pre-vaccination (Day 0 to Day 28) and post-vaccination (Day 28 to Day 56). A linear mixed model was used to assess the differences of outcomes at visits, and differences in change of outcomes between visits, with fixed effects being study group and visit. Each linear mixed model included the baseline value of the outcome as a covariate. A pairwise comparison of estimated marginal means was used to assess within-group changes for each group.

Analyses are reported for the intent-to-treat (ITT) population which included all participants who received product and had any post-randomization efficacy information available. Statistical analyses were completed using the R Statistical Software Package (Version 3.6.3 or newer) for Microsoft Windows (33). Tests of significance were performed as one-sided for primary outcomes, and two-sided for secondary outcomes at alpha level = 0.05. All data are presented as mean ± standard deviation (SD) unless otherwise specified.

## 3. Results

### 3.1. Study population

From the full clinical trial, 108 interested individuals were consented and screened and a total of 75 eligible participants were enrolled. In the ITT population, 50 participants were in the UP360 and Placebo groups (n = 25/group). One participant in each group was dropped from the study due to being booked for their COVID-19 vaccine, and one participant in the UP360 group was dropped as they tested positive for COVID-19 between baseline and Day 28. The mean compliance was 96.7 and 98.8% for UP360 and Placebo groups, respectively. The participant disposition figure is previously provided in Lewis et al., 2022 (16).

Participants were between 40 and 79 years of age and composed of 64% females in both groups. The majority of participants were college-educated or higher and employed full-time (Table 1). Demographics were not different between UP360 and Placebo groups.

**Table 1:** Baseline demographic for participants (*n* = 50).

Parameter	UP360*N* (%)		Placebo*N* (%)		*p*-value
Gender					0.943
Female	16	(64.0)	16	(64.0)	
Male	9	(36.0)	9	(36.0)	
Age, Mean (SD)	25	49.8 (6.9)	25	53.8 (9.5)	0.203
Marital Status					0.873
Married or Common-law	16	(64.0)	19	(76.0)	
Divorced or Separated	4	(16.0)	3	(12.0)	
Single	5	(20.0)	3	(12.0)	
Education level					0.149
Elementary/primary school	2	(8.0)	0	(0.0)	
High School graduate or GED	6	(24.0)	5	(20.0)	
College diploma	10	(40.0)	10	(40.0)	
University degree	2	(8.0)	7	(28.0)	
Master’s degree or higher	4	(16.0)	2	(8.0)	
Other	1	(4.0)	1	(4.0)	
Employment					0.121
Full-time employment	16	(64.0)	11	(44.0)	
Part-time employment	1	(4.0)	5	(20.0)	
Unemployed	1	(4.0)	3	(12.0)	
Retired	3	(12.0)	5	(20.0)	
Other	4	(16.0)	1	(4.0)	

All values presented are mean ± standard deviation (SD) unless otherwise stated.

### 3.2. Immune cell phenotypes

**Figure 1:**
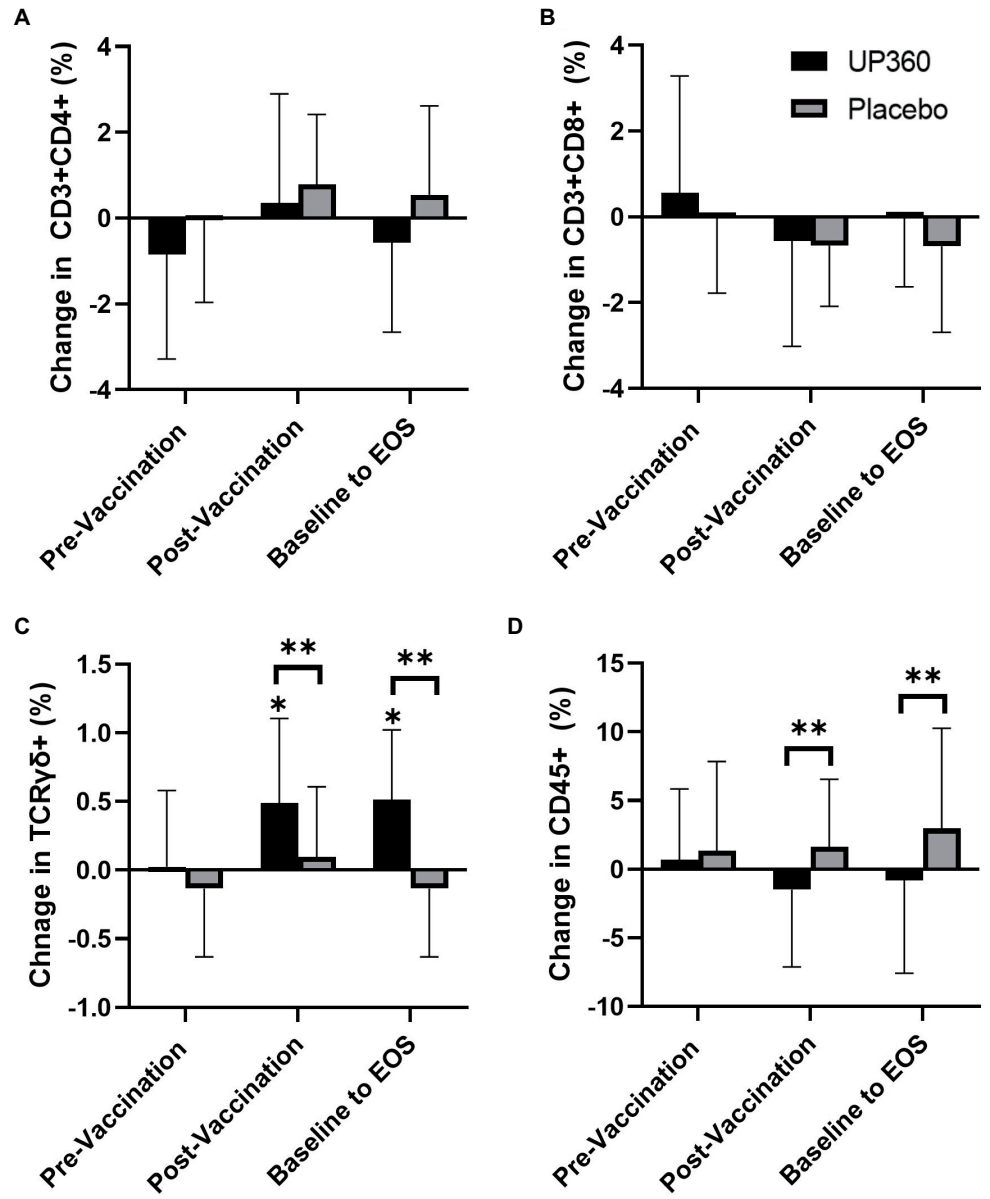
Change in the percentage of **(A)** Helper T cells (CD3 + CD4+), **(B)** Cytotoxic T Cells (CD3 + CD8+), **(C)** T-Cell Receptor Gamma Delta Cells (TCRγδ+), and **(D)** Total Lymphocytes (CD45+) between UP360 and Placebo in the pre-vaccination period (baseline to Day 28), post-vaccination period (Day 28 to Day 56) and from baseline to end-of-study (EOS, Day 56) in the ITT population (*n* = 50). Values presented are mean ± standard deviation (SD). *indicates a significant difference within-group at the specified timepoint and **indicates a significant difference between-groups.

There were no differences in the percentage of CD3 + CD4+ and CD3 + CD8+ populations between UP360 and Placebo (Figures 1A,B). There was a significantly higher percentage of TCRγδ+ cells in participants supplemented with UP360 compared to those on Placebo at Day 56, both from baseline and in the post-vaccination period (Figure 1C). The UP360 group had a reduction in the percentage of CD45+ cells in the post-vaccination period, and from baseline at Day 56 compared to Placebo (*p* ≤ 0.044) (Figure 1D). There was a reduction in NK cells (CD3-CD56+) from baseline at Day 56 in participants on Placebo (*p* = 0.025) (data not shown, reported previously (16)).

### 3.3. Total and vaccine-specific immunoglobulin response

Participants on Placebo had a significant increase in levels of IgG in the pre-vaccination period (Table 2). However, there were no significant changes in total IgA, IgG or IgM levels with UP360 supplementation after 56 days.

**Table 2:** Total immunoglobulin and influenza-B specific immunoglobulin concentrations in blood of participants (*n* = 50).

Immunoglobulin	Study timepoint	UP360 mean ± SD within group *p*-value	Placebo mean ± SD within group *p*-value	UP360 vs. Placebo
Total IgA (g/L)	Baseline	2.161 ± 0.973	2.145 ± 0.808	0.994
	Day 28	2.234 ± 0.990	2.217 ± 0.859	0.347
	Day 56	2.214 ± 0.987	2.190 ± 0.873	0.487
	Pre-vaccination (Baseline to Day 28)	0.013 ± 0.124	0.043 ± 0.118	0.431
		0.698	0.14	
	Post-vaccination (Day 28 to Day 56)	−0.020 ± 0.094	−0.027 ± 0.178	0.844
		0.518	0.351	
	Baseline to Day 56	−0.007 ± 0.131	0.016 ± 0.175	0.56
		0.796	0.585	
Total IgG (g/L)	Baseline	9.789 ± 2.057	10.865 ± 1.512	0.871
	Day 28	10.153 ± 2.166	11.112 ± 1.674	0.639
	Day 56	10.010 ± 1.990	11.034 ± 1.659	0.391
	Pre-vaccination (Baseline to Day 28)	0.208 ± 0.508	0.236 ± 0.560	0.664
		0.076	0.031	
	Post-vaccination (Day 28 to Day 56)	−0.143 ± 0.414	−0.078 ± 0.457	0.338
		0.246	0.506	
	Baseline to Day 56	0.065 ± 0.443	0.158 ± 0.550	0.434
		0.536	0.132	
Total IgM (g/L)	Baseline	1.056 ± 0.436	1.260 ± 1.170	0.992
	Day 28	1.107 ± 0.455	1.323 ± 1.304	0.472
	Day 56	1.086 ± 0.445	1.302 ± 1.182	0.464
	Pre-vaccination (Baseline to Day 28)	0.016 ± 0.078	0.039 ± 0.151	0.512
		0.474	0.133	
	Post-vaccination (Day 28 to Day 56)	−0.022 ± 0.081	−0.020 ± 0.170	0.917
		0.447	0.456	
	Baseline to Day 56	−0.006 ± 0.067	0.018 ± 0.119	0.505
		0.964	0.447	
Influenza B IgA (Standard Units)	Baseline	4 ± 2.6	3.4 ± 2.7	0.7561
	Day 28	4 ± 3.3	3.5 ± 2.6	0.9848
	Day 56	3.8 ± 2.4	3.9 ± 3.1	0.3753
	Pre-vaccination (Baseline to Day 28)	−0.1 ± 3.3	± 2.7	0.9236
		0.9023	0.8371	
	Post-vaccination (Day 28 to Day 56)	−0.1 ± 3.1	0.4 ± 3.4	0.6308
		0.8598	0.4896	
	Baseline to Day 56	−0.2 ± 2.2	± 1.8	0.4741
		0.7646	0.3704	
Influenza B IgG (Standard Units)	Baseline	12.2 ± 3.4	13.1 ± 4.5	0.9123
	Day 28	10.9 ± 3.6	12.8 ± 4.9	0.0677
	Day 56	12.8 ± 4.1	14.1 ± 4.8	0.4083
	Pre-vaccination (Baseline to Day 28)	−1.3 ± 1.5	−0.3 ± 2.1	0.7188
		0.0193	0.5862	
	Post-vaccination (Day 28 to Day 56)	1.9 ± 2.2	1.3 ± 3.0	0.5382
		0.0006	0.0141	
	Baseline to Day 56	0.6 ± 2.3	1.0 ± 2.9	0.6978
		0.2566	0.0541	
Influenza B IgM (Standard Units)	Baseline	2 ± 1.8	1.8 ± 2.5	0.9896
	Day 28	1.9 ± 1.8	1.9 ± 2.7	0.5615
	Day 56	2.7 ± 3.2	3.3 ± 5.5	0.1024
	Pre-vaccination (Baseline to Day 28)	−0.2 ± 0.6	0.2 ± 0.8	0.6639
		0.7721	0.77	
	Post-vaccination (Day 28 to Day 56)	0.8 ± 1.8	1.3 ± 4.8	0.4309
		0.1464	0.0107	
	Baseline to Day 56	0.6 ± 1.8	1.5 ± 4.8	0.2321
		0.2439	0.0046	

All values presented are mean ± standard deviation (SD) unless otherwise stated.

For influenza specific immunoglobulins, there were no significant differences in influenza B specific-IgA, IgG or IgM between UP360 and Placebo. However in the post-vaccination period, participants supplemented with UP360 had a significant increase in influenza B-specific IgG levels (Table 2). There were significant increases in levels of influenza B-specific IgM in the post-vaccination period and from baseline to Day 56 in the Placebo group.

There were no differences in influenza A specific-IgA, IgG or IgM levels with UP360 supplementation compared to Placebo (data not shown). As previously reported (16), the Placebo group had a decrease in influenza A-specific IgG in the pre-vaccination period (*p* = 0.0048) and increases in levels of influenza A-specific IgM in the post-vaccination period and from baseline to Day 56 (*p* ≤ 0.0252).

### 3.4. Serum cytokines and glutathione peroxidase

**Figure 2:**
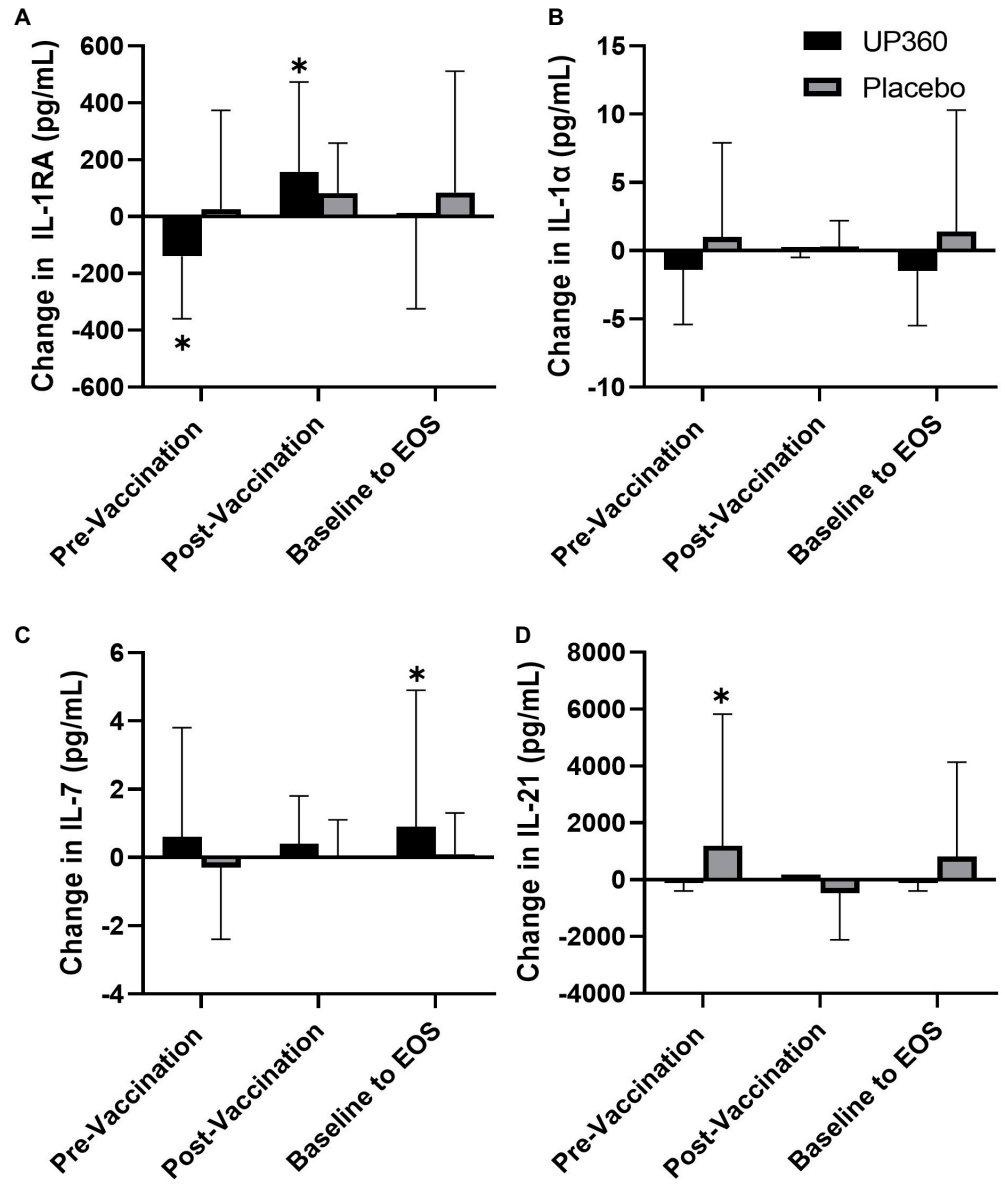
Change in the serum concentration of **(A)** IL-1RA, **(B)** IL-1α, **(C)** IL-7, and **(D)** IL-21 between UP360 and Placebo in the pre-vaccination period (baseline to Day 28), post-vaccination period (Day 28 to Day 56) and from baseline to end-of-study (EOS, Day 56) in the ITT population (*n* = 50). Values presented are mean ± standard deviation (SD). *indicates a significant difference within-group at the specified timepoint.

Participants supplemented with UP360 had a decrease in IL-1RA concentration in the pre-vaccination period (Figures 2A, *p* = 0.0381). However, in the post-vaccination period, IL-1RA increased in this group (Figures 2A, *p* = 0.0482). Serum levels of IL-7 were increased from baseline at Day 56 with UP360 supplementation (Figures 2C, *p* = 0.0458). Participants on Placebo had an increase in IL-21 in the pre-vaccination period (Figures 2D, *p* = 0.007) but there were no other differences in serum cytokines observed during the study. There was a significant increase in GSH-Px from 107.8 ± 31.0 mU/ml at baseline to 122.3 ± 11.9 mU/ml at Day 28 with UP360 supplementation (*p* = 0.0214). There was no significant change in GSH-Px during the study for participants on Placebo.

### 3.5. Upper respiratory tract infections

Mean global severity index was not significantly different between UP360 and Placebo groups in the pre-vaccination compared to post-vaccination period (Supplementary Table 3). There were no differences in mean symptom severity index, URTI symptom frequency, duration or severity, or the use of cold and flu medication between groups during the study period. The mean number of well days ranged from 97.5–98.2% for participants supplemented with UP360 and 98.5–99.6% for those on Placebo in the pre-and post-vaccination periods.

### 3.6. Quality of life

There was no difference on measures of the impact of COVID-19 on QoL from participants supplemented with UP360 compared to those on Placebo (Table 3). Vitality and QoL were not significantly different between UP360 and Placebo groups in the pre-vaccination period, post-vaccination period, or from baseline at Day 56 (Supplementary Table 4).

**Table 3:** Impact of COVID-19 on participant’s quality of life (QoL) assessed by the COVID-19 impact on QoL questionnaire (ITT) (*n* = 50).

Question	Study timepoint	UP360 mean ± SD within group *p*-value	Placebo mean ± SD within group *p*-value	UP360 vs. Placebo
I think my quality of life is lower than before	Baseline	2.9 ± 1.4	2.8 ± 1.3	1
	Day 28	2.7 ± 1.4	2.7 ± 1.2	1
	Day 56	2.8 ± 1.4	2.6 ± 1.4	0.999
	Pre-vaccination (Baseline to Day 28)	−0.2 ± 1.1	−0.2 ± 0.9	1
		0.992	0.998	
	Post-vaccination (Day 28 to Day 56)	0.2 ± 0.9	−0.1 ± 0.8	1
		0.998	1	
	Baseline to Day 56	−0.0 ± 0.9	−0.3 ± 1.2	1
		1	0.934	
I think my mental health has deteriorated	Baseline	2.3 ± 1.2	2.4 ± 1.2	1
	Day 28	2.2 ± 1.2	2.0 ± 1.1	0.998
	Day 56	2.2 ± 1.2	2.3 ± 1.2	1
	Pre-vaccination (Baseline to Day 28)	−0.0 ± 0.6	−0.4 ± 1.0	1
		1	0.546	
	Post-vaccination (Day 28 to Day 56)	−0.0 ± 0.7	0.3 ± 1.0	1
		1	0.827	
	Baseline to Day 56	−0.1 ± 0.4	−0.1 ± 1.0	1
		1	1	
I think my physical health may deteriorate	Baseline	2.3 ± 1.2	2.4 ± 1.2	1
	Day 28	2.2 ± 1.2	2.0 ± 1.1	0.998
	Day 56	2.2 ± 1.2	2.3 ± 1.2	1
	Pre-vaccination (Baseline to Day 28)	−0.0 ± 0.6	−0.4 ± 1.0	1
		1	0.546	
	Post-vaccination (Day 28 to Day 56)	−0.0 ± 0.7	0.3 ± 1.0	1
		1	0.827	
	Baseline to Day 56	−0.1 ± 0.4	−0.1 ± 1.0	1
		1	1	
I feel more tense than before	Baseline	2.6 ± 1.3	2.6 ± 1.0	1
	Day 28	2.6 ± 1.3	2.3 ± 1.1	0.998
	Day 56	2.5 ± 1.3	2.5 ± 1.1	1
	Pre-vaccination (Baseline to Day 28)	−0.0 ± 1.2	−0.3 ± 1.1	1
		1	0.918	
	Post-vaccination (Day 28 to Day 56)	−0.1 ± 0.9	0.2 ± 1.2	1
		1	0.989	
	Baseline to Day 56	−0.1 ± 1.1	−0.1 ± 1.1	1
		1	1	
I feel more depressed than before	Baseline	2.1 ± 1.2	2.0 ± 0.9	1
	Day 28	2.4 ± 1.4	2.0 ± 0.9	0.987
	Day 56	2.5 ± 1.4	2.3 ± 1.1	1
	Pre-vaccination (Baseline to Day 28)	0.2 ± 0.7	0.1 ± 1.0	1
		0.963	1	
	Post-vaccination (Day 28 to Day 56)	0.1 ± 1.1	0.2 ± 1.1	1
		1	0.963	
	Baseline to Day 56	0.4 ± 1.2	0.3 ± 1.3	1
		0.718	0.829	
I feel that my personal safety is at risk	Baseline	1.9 ± 0.9	1.8 ± 1.0	1
	Day 28	1.8 ± 1.0	2.0 ± 0.9	0.996
	Day 56	2.2 ± 1.2	2.0 ± 1.0	0.996
	Pre-vaccination (Baseline to Day 28)	−0.1 ± 0.8	0.2 ± 1.4	1
		1	0.991	
	Post-vaccination (Day 28 to Day 56)	0.4 ± 1.0	−0.1 ± 1.4	1
		0.653	1	
	Baseline to Day 56	0.3 ± 0.9	0.1 ± 1.0	1
		0.865	1	
Total score	Baseline	14.2 ± 5.6	14.0 ± 4.7	1
	Day 28	14.1 ± 6.5	13.3 ± 4.4	1
	Day 56	14.6 ± 6.4	13.9 ± 5.6	1
	Pre-vaccination (Baseline to Day 28)	−0.1 ± 3.4	−0.7 ± 3.8	1
		1	0.995	
	Post-vaccination (Day 28 to Day 56)	0.6 ± 3.5	0.6 ± 4.1	1
		0.999	0.999	
	Baseline to Day 56	0.5 ± 3.7	−0.1 ± 5.1	1
		1	1	

All values presented are mean ± standard deviation (SD) unless otherwise stated.

### 3.7. Safety

Supplementation with UP360 for 56 days was found to be safe and well tolerated by participants. There were eight AEs reported by seven participants in the UP360 group. One participant, who also tested positive for COVID-19, reported tiredness, and was removed from the study. Of the remaining seven AEs, there were two reports of tiredness, and one report each of vomiting, cold symptoms, feeling sick, muscle soreness, and stomach cramps. There were a total of six AEs reported by six participants in the Placebo group: one report each of nausea, headache, heart burn, stomachache, increase in ALT, and hot flashes. All AEs were deemed ‘unlikely’ or ‘not related’ to the study products except for one report of stomach cramps in the UP360 group that was categorized as ‘possible’. All AEs had resolved by the end of the study.

There were no significant changes in clinical chemistry measures or vitals during the study with UP360 supplementation (data not shown). All clinical chemistry values outside the normal laboratory range were deemed not clinically relevant by the MD with the exception of potassium, AST, and ALT, in the Placebo group and has been previously described (16).

## 4. Discussion

*Aloe vera* gel powder, rosemary and *Poria cocos* extracts have been reported to have antioxidant and anti-inflammatory properties (17–20), (24), inhibit influenza viral replication (17), and increase Ig production (23) and antibody titers following immunization (34). To date there have been no studies that have examined the immunomodulatory effects of these ingredients in a nutraceutical formulation, in randomized, double-blind, placebo-controlled clinical trials.

Supplementation with UP360 for 56 days was found to significantly increase the percentage of TCR gamma-delta (γδ) cells, known as γδ T cells, in the 28-day post-vaccination period compared to Placebo. Further, UP360 supplementation significantly increased IL-7 levels over the 56-day study period. This increase in IL-7 may have been involved in the expansion of the γδ T cell population as IL-7 has been shown to control the homeostasis and promotion of γδ T cells (35, 36). γδ T cells are a specialized subset of T cells largely present at many entry points in the body, including gastrointestinal and respiratory tracts. Early in their development, these cells migrate and persist as resident cells in these areas. Primarily considered innate-like lymphocytes, they can develop memory-like adaptive responses which connects the two arms of the immune system (37). Based on their strategic anatomical locations and innate-like responses in killing infected cells, recruiting other immune cells, phagocytosis activation, and limiting pathogen translocation, γδ T cells often provide a first line of immune defense. Further, due to their adaptive immune cell nature, γδ T cells also undergo rapid population expansion and provide pathogen-specific protection with secondary immune challenges. Besides promoting the growth of dendritic cells as antigen presenting cells (APCs), these cells could directly activate the adaptive immune response by quickly serving as APCs (38, 39). In general, with respect to the immune system, the γδ T cells function to provide protective immunity against pathogens, immunosurveillance, and modulation of innate and adaptive immune responses for a heightened rapid and effective immune response (40–42). In humans, γδ T cells constitute less than 10% of peripheral blood T cells, yet *in vitro* and animal models have demonstrated that these cells exert direct effects on influenza infection through elimination of the virus (43, 44), and inhibition of viral replication through production of interferon (IFN)-γ (45, 46). A randomized, double-blind, placebo-controlled study demonstrated that supplementation with an encapsulated concentrate of fruits and vegetables increased circulating γδ T cells compared to Placebo in healthy people (31). Similarly, supplementation with *Camellia sinensis* for 3 months has been reported to increase γδ T cell proliferation and IFN-γ, production in healthy adults (47). These studies support the observation in the current study that nutraceuticals have a role in modulating this lymphocyte subset.

Further, as previously discussed, the individual ingredients of UP360 have been shown to have anti-inflammatory and antioxidant effects (17–20), (24). Supplementation with UP360 increased GSH-Px, an enzyme important for neutralizing free radicals in the pre-vaccination period, as well as increased IL-1RA, an anti-inflammatory cytokine, in the post-vaccination period. In a rodent study, administration of IL-1RA has been shown to significantly improve clinical scores and survival following influenza challenge (48). This suggests UP360 supplementation may be beneficial in mitigating the oxidative stress response that occurs in response to vaccination.

While an increase in the TCRγδ+ cell population was found, there was a significant reduction in the percentage of total lymphocytes (CD45+) in the post-vaccination period with UP360 supplementation compared to Placebo. The implications of this are unknown and warrant further investigation. However, it is noteworthy that despite this reduction in total lymphocyte population, UP360 supplementation promoted the expansion of a specific subset of the lymphocyte population, γδ + T cells, which is relevant to supporting immune health, specifically in the context of influenza infection.

In addition to the effect on T lymphocytes, UP360 supplementation increased influenza B-specific IgG concentrations in the 28-day post-vaccination period. It has been reported that γδ T cells help B cells produce different classes of antibodies through the secretion inflammatory molecules including IFN-γ, granulocyte–macrophage colony stimulating factor (GM-CSF) and tumor necrosis factor (TNF) (49–52). *In vitro* and animal models have demonstrated that components of rosemary increase IgM and IgG production following immune challenges (23). It is possible the components of UP360 are having both direct and indirect effects on Ig production and γδ T cells and playing a role in the increased production of IgG from B cells. IgG comprises approximately 75% of serum antibodies (53) and is responsible for the majority of humoral protective immunity induced by influenza infection (30). It is possible that the improved antibody response following vaccination is a result of the promotion of γδ T cell activity as IgG is indicative of T helper cell functionality (13). This potential mechanism of action warrants exploration in future studies.

In the current study, there were no differences in URTI symptom duration, severity or frequency, consistent with previous findings with UP460 supplementation, a formulation containing *A. catechu and S. baicalensis* (16). Further, there were no significant changes in vitality and QoL in the pre-and post-vaccination periods. As there is a strong relationship between physical wellbeing and mental health (54–56), the impact of COVID-19 on QoL was assessed to understand the impact of the pandemic on participants’ well-being as a potential confounder. There were no significant changes in QoL due to COVID-19. However, there were four participants supplemented with UP360 that reported they “agreed” or “strongly agreed” with feelings of lower QoL, physical and mental health deterioration, being more tense and depressed than before the COVID-19 pandemic. While none of these four participants reported being ill, the connection between mental and physical wellbeing should be examined further as a potential confounder in future studies.

The safety of UP360 was evaluated using incidence of AEs, clinical chemistry parameters and vitals. All AEs were resolved by the end of the study period. There were no statistically significant or clinically relevant changes in clinical chemistry parameters or vitals and provided evidence of a good safety profile for UP360 during the 56-day study period.

While results of this study are promising, the incidence of cold and flu was very low, likely the result of the study being conducted in the 2021 winter season during the COVID-19 pandemic. From the perspective of public health this was beneficial, it was challenging to differentiate the efficacy of UP360 on cold and flu like symptoms during what is considered ‘flu season’. This was an unavoidable study limitation. Further, the current study examined circulating γδ T cells, however these cells are also found in the respiratory tract for which lung-specific γδ T cells have been identified and possess distinct immunoregulatory functions (57–59). This subset of lung-specific γδ T cells were not examined.

The results of this study suggest several areas for future research. As UP360 supplementation influenced γδ T cells, future research is warranted to examine the effects of UP360 supplementation both on circulating and lung-specific γδ T cells when exploring their potential mechanism of action. Further, the implications on immune function due to the changes on total lymphocytes with UP360 supplementation warrants further investigation. Future studies should consider measuring both systemic and lung-specific T cell subsets, as well as their interaction. Further, as mentioned above there is a strong connection between mental and physical wellbeing and future studies should consider examining additional factors that may have an influenced an individuals’ immune response.

In conclusion, this study showed that supplementation with UP360 significantly increased the percentage of γδ + T cells and levels of influenza B-specific IgG in the post-vaccination period. This suggests UP360 supplementation was efficacious in producing a robust, healthy mucosal immune response following influenza vaccination. Further, no changes in subjective measures of URTI illness or QoL demonstrates there was no negative impact by UP360 on participants during the 56-day supplementation period. There were no clinically relevant changes in clinical chemistry, vitals or AEs during the study establishing the safety profile of UP360 supplementation with and without vaccination.

## Data availability statement

The original contributions presented in the study are included in the article/Supplementary material, further inquiries can be directed to the corresponding author.

## Ethics statement

This study involving human participants was reviewed and approved by Institutional Review Board (IRB) Services. The patients/participants provided their written informed consent to participate in this study.

## Author contributions

EL, DC, NG, and ME contributed to the conception and design of the study. EL, DC, and ME conducted the study, collected, and compiled data. EL and ME interpreted the data, and drafted the manuscript. DC and ME supervised. All authors contributed to the article and approved the submitted version.

## Funding

This research was funded by Unigen Inc. (2121 South State Street Suite 400, Tacoma, Washington 98405 USA). Unigen was involved in the design of the study and reviewed the manuscript but was not involved in conduct of the study, analysis of data, or interpretation of findings.

## Conflict of interest

All authors were employed by the company KGK Science Inc. The authors declare that this study received funding from Unigen Inc. The funder was involved in the study design and reviewed the manuscript but was not involved in the collection, analysis, or interpretation of data.

## Publisher’s note

All claims expressed in this article are solely those of the authors and do not necessarily represent those of their affiliated organizations, or those of the publisher, the editors and the reviewers. Any product that may be evaluated in this article, or claim that may be made by its manufacturer, is not guaranteed or endorsed by the publisher.

## Footnotes

^1^ www.randomization.com

## References

[1] National Institute on Ageing. “The underappreciated burden of inuenza amongst Canada’s older population,” in And what we need to do about it. Toronto, ON: National Institute on Ageing White Paper (2018).

[2] Schanzer DL Sevenhuysen C Winchester B Mersereau T. Estimating influenza deaths in Canada, 1992–2009. PLoS One. (2013) 8:e80481. doi: 10.1371/journal.pone.0080481, PMID: 24312225 PMC3842334

[3] Schanzer DL McGeer A Morris K. Statistical estimates of respiratory admissions attributable to seasonal and pandemic influenza for Canada. Influenza Other Respir Viruses. (2013) 7:799–808. doi: 10.1111/irv.12011, PMID: 23122189 PMC3796862

[4] Department of Family Medicine and Community Health. Wisconsin Upper Respiratory System Survey (WURSS). Madison, Wisconsin, United States: University of Wisconsin (2019). Available at: https://www.fammed.wisc.edu/wurss/

[5] Richardson S Hirsch JS Narasimhan M Crawford JM McGinn T Davidson KW. Presenting characteristics, comorbidities, and outcomes among 5700 patients hospitalized with COVID-19 in the new York City area. JAMA. (2020) 323:2052–9. doi: 10.1001/jama.2020.6775, PMID: 32320003 PMC7177629

[6] Chen N Zhou M Dong X Qu J Gong F Han Y. Epidemiological and clinical characteristics of 99 cases of 2019 novel coronavirus pneumonia in Wuhan, China: a descriptive study. Lancet. (2020) 395:507–13. doi: 10.1016/S0140-6736(20)30211-7, PMID: 32007143 PMC7135076

[7] Zanettini C Omar M Dinalankara W Imada EL Colantuoni E Parmigiani G. Influenza Vaccination and COVID19 Mortality in the USA. Version 1. medRxiv. Preprint. (2020). doi: 10.1101/2020.06.24.20129817

[8] CDC. (2020). 21 flu season summary FAQ: Centers for Disease Control and Prevention; 2021. Available from: https://www.cdc.gov/flu/season/faq-flu-season-2020-2021.htm.

[9] Dawood FS Chung JR Kim SS Zimmerman RK Nowalk MP Jackson ML. Interim estimates of 2019–20 seasonal influenza vaccine effectiveness—United States, February 2020. MMWR Morb Mortal Wkly Rep. (2020) 69:177–82. doi: 10.15585/mmwr.mm6907a1, PMID: 32078591 PMC7043386

[10] Flannery B Kondor RJG Chung JR Gaglani M Reis M Zimmerman RK. Spread of antigenically drifted influenza a(H3N2) viruses and vaccine effectiveness in the United States during the 2018–2019 season. J Infect Dis. (2019) 221:8–15. doi: 10.1093/infdis/jiz543 PMC7325528 31665373

[11] Pae M Meydani SN Wu D. The role of nutrition in enhancing immunity in aging. Aging Dis. (2012) 3:91–129. PMID: 22500273 PMC3320807

[12] Yeh T-L Shih P-C Liu S-J Lin C-H Liu J-M Lei W-T. The influence of prebiotic or probiotic supplementation on antibody titers after influenza vaccination: a systematic review and meta-analysis of randomized controlled trials. Drug Des Devel Ther. (2018) 12:217–30. doi: 10.2147/DDDT.S155110, PMID: 29416317 PMC5790137

[13] Rizzardini G Eskesen D Calder PC Capetti A Jespersen L Clerici M. Evaluation of the immune benefits of two probiotic strains Bifidobacterium animalis ssp. lactis, BB-12® and lactobacillus paracasei ssp. paracasei, L. casei 431® in an influenza vaccination model: a randomised, double-blind, placebo-controlled study. Br J Nutr. (2012) 107:876–84. doi: 10.1017/S000711451100420X, PMID: 21899798

[14] Xiao L Engen PA Leusink-Muis T van Ark I Stahl B Overbeek SA. The combination of 2'-Fucosyllactose with short-chain Galacto-oligosaccharides and long-chain Fructo-oligosaccharides that enhance influenza vaccine responses is associated with mucosal immune regulation in mice. J Nutr. (2019) 149:856–69. doi: 10.1093/jn/nxz006, PMID: 31050747 PMC6499104

[15] Fernández-Ferreiro A Formigo-Couceiro FJ Veiga-Gutierrez R Maldonado-Lobón JA Hermida-Cao AM Rodriguez C. Effects of Loigolactobacillus coryniformis K8 CECT 5711 on the immune response of elderly subjects to COVID-19 vaccination: a randomized controlled trial. Nutrients. (2022) 14:228. doi: 10.3390/nu14010228, PMID: 35011103 PMC8747230

[16] Lewis ED Crowley DC Guthrie N Evans M. Role of Acacia catechu and Scutellaria baicalensis in enhancing immune function following influenza vaccination of healthy adults: a randomized, triple-blind, placebo-controlled clinical trial. J Am Nutr Assoc. (2022):1–13. doi: 10.1080/27697061.2022.2145525, PMID: 36413261

[17] Sun Z Yu C Wang W Yu G Zhang T Zhang L. Aloe polysaccharides inhibit influenza a virus infection-a promising natural anti-flu drug. Front Microbiol. (2018) 9:2338. doi: 10.3389/fmicb.2018.02338, PMID: 30319596 PMC6170609

[18] Yagi A Byung PY. Immune modulation of Aloe vera: acemannan and gut microbiota modulator. J Gastroenterol Hepatol Res. (2015) 4:1707–21. doi: 10.17554/j.issn.2224-3992.2015.04.525

[19] Kumar S Tiku AB. Immunomodulatory potential of acemannan (polysaccharide from Aloe vera) against radiation induced mortality in Swiss albino mice. Food Agric Immunol. (2016) 27:72–86. doi: 10.1080/09540105.2015.1079594

[20] Langmead L Makins RJ Rampton DS. Anti-inflammatory effects of aloe vera gel in human colorectal mucosa in vitro. Aliment Pharmacol Ther. (2004) 19:521–7. doi: 10.1111/j.1365-2036.2004.01874.x, PMID: 14987320

[21] Song E-J Españo E Nam J-H Kim J Shim K-S Shin E. Adjuvanticity of processed Aloe vera gel for influenza vaccination in mice. Immune Network. (2020) 20:e31. doi: 10.4110/in.2020.20.e31 32895618 PMC7458799

[22] Ahmed HM Babakir-Mina M. Investigation of rosemary herbal extracts (Rosmarinus officinalis) and their potential effects on immunity. Phytother Res. (2020) 34:1829–37. doi: 10.1002/ptr.6648, PMID: 32086980

[23] Al Sheyab FM Abuharfeil N Salloum L Bani Hani R Awad DS. The effect of rosemary Rosmarinus officinalis. L plant extracts on the immune response and lipid profile in mice. J. Biol. Life Sci. (2011) 3:37–58. doi: 10.5296/jbls.v3i1.906

[24] Sun Y. Biological activities and potential health benefits of polysaccharides from Poria cocos and their derivatives. Int J Biol Macromol. (2014) 68:131–4. doi: 10.1016/j.ijbiomac.2014.04.010, PMID: 24751506

[25] Wu Y Li S Li H Zhao C Ma H Zhao X. Effect of a polysaccharide from Poria cocos on humoral response in mice immunized by H1N1 influenza and HBsAg vaccines. Int J Biol Macromol. (2016) 91:248–57. doi: 10.1016/j.ijbiomac.2016.05.046, PMID: 27185068

[26] Zhang W Cheng N Wang Y Zheng X Zhao Y Wang H. Adjuvant activity of PCP-II, a polysaccharide from Poria cocos, on a whole killed rabies vaccine. Virus Res. (2019) 270:197638. doi: 10.1016/j.virusres.2019.06.001, PMID: 31173772

[27] Moher D Hopewell S Schulz KF Montori V Gotzsche PC Devereaux PJ. CONSORT 2010 explanation and elaboration: updated guidelines for reporting parallel group randomised trials. Int J Surg. (2012) 10:28–55. doi: 10.1016/j.ijsu.2011.10.001, PMID: 22036893

[28] Barrett B Brown RL Mundt MP Thomas GR Barlow SK Highstrom AD. Validation of a short form Wisconsin upper respiratory symptom survey (WURSS-21). Health Qual Life Outcomes. (2009) 7:76. doi: 10.1186/1477-7525-7-76, PMID: 19674476 PMC2748069

[29] Repišti S Jovanović N IMPULSE study group (2020). COVID-19–impact on quality of life (COV19-QoL) scale v1.5. Available at: https://www.researchgate.net/publication/34089 9554 _COVID-19-Impact_on_Quality_of_Life_ COV19-QoL_scale_v15.

[30] Olivares M Diaz-Ropero MP Sierra S Lara-Villoslada F Fonolla J Navas M. Oral intake of lactobacillus fermentum CECT5716 enhances the effects of influenza vaccination. Nutrition. (2007) 23:254–60. doi: 10.1016/j.nut.2007.01.004, PMID: 17352961

[31] Nantz MP Rowe CA Nieves C Jr Percival SS. Immunity and antioxidant capacity in humans is enhanced by consumption of a dried, encapsulated fruit and vegetable juice concentrate. J Nutr. (2006) 136:2606–10. doi: 10.1093/jn/136.10.2606, PMID: 16988134

[32] De Vrese M Winkler P Rautenberg P Harder T Noah C Laue C. Probiotic bacteria reduced duration and severity but not the incidence of common cold episodes in a double blind, randomized, controlled trial. Vaccine. (2006) 24:6670–4. doi: 10.1016/j.vaccine.2006.05.048 16844267

[33] R Core Team. R: A Language and Environment for Statistical Computing. Vienna, Austria: R Foundation for Statistical Computing (2019).

[34] Sunil MA Sunitha VS Radhakrishnan EK Jyothis M. Immunomodulatory activities of Acacia catechu, a traditional thirst quencher of South India. Journal of Ayurveda and Integrative Medicine. (2019) 10:185–91. doi: 10.1016/j.jaim.2017.10.010, PMID: 29502869 PMC6822161

[35] Michel M-L Pang DJ Haque SFY Potocnik AJ Pennington DJ Hayday AC. Interleukin 7 (IL-7) selectively promotes mouse and human IL-17-producing γδ cells. Proc Natl Acad Sci U S A. (2012) 109:17549–54. doi: 10.1073/pnas.1204327109, PMID: 23047700 PMC3491488

[36] Baccala R Witherden D Gonzalez-Quintial R Dummer W Surh CD Havran WL. Gamma delta T cell homeostasis is controlled by IL-7 and IL-15 together with subset-specific factors. J Immunol. (2005) 174:4606–12. doi: 10.4049/jimmunol.174.8.4606, PMID: 15814683

[37] Sabbaghi A Miri SM Keshavarz M Mahooti M Zebardast A Ghaemi A. Role of γδ T cells in controlling viral infections with a focus on influenza virus: implications for designing novel therapeutic approaches. Virol J. (2020) 17:174. doi: 10.1186/s12985-020-01449-0, PMID: 33183352 PMC7659406

[38] Lawand M Déchanet-Merville J Dieu-Nosjean M-C. Key features of Gamma-Delta T-cell subsets in human diseases and their immunotherapeutic implications. Front Immunol. (2017) 8. doi: 10.3389/fimmu.2017.00761, PMID: 28713381 PMC5491929

[39] Brandes M Willimann K Moser B. Professional antigen-presentation function by human gammadelta T cells. Science. (2005) 309:264–8. doi: 10.1126/science.1110267, PMID: 15933162

[40] Ribot JC Lopes N Silva-Santos B. γδ T cells in tissue physiology and surveillance. Nat Rev Immunol. (2021) 21:221–32. doi: 10.1038/s41577-020-00452-4 33057185

[41] Holderness J Schepetkin IA Freedman B Kirpotina LN Quinn MT Hedges JF. Polysaccharides isolated from Açaí fruit induce innate immune responses. PLoS One. (2011) 6:e17301. doi: 10.1371/journal.pone.0017301, PMID: 21386979 PMC3046208

[42] Yazdanifar M Mashkour N Bertaina A. Making a case for using γδ T cells against SARS-CoV-2. Crit Rev Microbiol. (2020) 46:689–702. doi: 10.1080/1040841X.2020.1822279, PMID: 33023358

[43] Li H Xiang Z Feng T Li J Liu Y Fan Y. Human Vγ9Vδ2-T cells efficiently kill influenza virus-infected lung alveolar epithelial cells. Cell Mol Immunol. (2013) 10:159–64. doi: 10.1038/cmi.2012.70, PMID: 23353835 PMC4003054

[44] Qin G Mao H Zheng J Sia SF Liu Y Chan PL. Phosphoantigen-expanded human gammadelta T cells display potent cytotoxicity against monocyte-derived macrophages infected with human and avian influenza viruses. J Infect Dis. (2009) 200:858–65. doi: 10.1086/605413, PMID: 19656068 PMC7110194

[45] Carding SR Allan W Kyes S Hayday A Bottomly K Doherty PC. Late dominance of the inflammatory process in murine influenza by gamma/delta + T cells. J Exp Med. (1990) 172:1225–31. doi: 10.1084/jem.172.4.1225, PMID: 2145388 PMC2188600

[46] Sant S Jenkins MR Dash P Watson KA Wang Z Pizzolla A. Human γδ T-cell receptor repertoire is shaped by influenza viruses, age and tissue compartmentalisation. Clin Transl Immunol. (2019) 8:e1079-e. doi: 10.1002/cti2.1079 PMC6756999 31559018

[47] Rowe CA Nantz MP Bukowski JF Percival SS. Specific formulation of Camellia sinensis prevents cold and flu symptoms and enhances gamma,delta T cell function: a randomized, double-blind, placebo-controlled study. J Am Coll Nutr. (2007) 26:445–52. doi: 10.1080/07315724.2007.10719634, PMID: 17914132

[48] Shirey KA Lai W Patel MC Pletneva LM Pang C Kurt-Jones E. Novel strategies for targeting innate immune responses to influenza. Mucosal Immunol. (2016) 9:1173–82. doi: 10.1038/mi.2015.141, PMID: 26813341 PMC5125448

[49] Paul S Lal G. Regulatory and effector functions of gamma-delta (γδ) T cells and their therapeutic potential in adoptive cellular therapy for cancer. Int J Cancer. (2016) 139:976–85. doi: 10.1002/ijc.30109, PMID: 27012367

[50] Rezende RM Lanser AJ Rubino S Kuhn C Skillin N Moreira TG. γδ T cells control humoral immune response by inducing T follicular helper cell differentiation. Nat Commun. (2018) 9:3151. doi: 10.1038/s41467-018-05487-9, PMID: 30089795 PMC6082880

[51] Caccamo N Battistini L Bonneville M Poccia F Fournié JJ Meraviglia S. CXCR5 identifies a subset of Vγ9Vδ2 T cells which secrete IL-4 and IL-10 and help B cells for antibody production. J Immunol. (2006) 177:5290–5. doi: 10.4049/jimmunol.177.8.5290, PMID: 17015714

[52] Inoue SI Niikura M Asahi H Kawakami Y Kobayashi F. γδ T cells modulate humoral immunity against plasmodium berghei infection. Immunology. (2018) 155:519–32. doi: 10.1111/imm.12997, PMID: 30144035 PMC6231001

[53] Schroeder HW Jr Cavacini L. Structure and function of immunoglobulins. J Allergy Clin Immunol. (2010) 125:S41–52. doi: 10.1016/j.jaci.2009.09.046, PMID: 20176268 PMC3670108

[54] Nabi H Kivimaki M De Vogli R Marmot MG. Singh-Manoux a, vol. 337. Positive and negative affect and risk of coronary heart disease: Whitehall II prospective cohort study. Bmj (2008). a118 p doi: 10.1136/bmj.a118 PMC2443594 18595926

[55] Surtees P Wainwright N Luben R Wareham N Bingham S Khaw K-T. Psychological distress, major depressive disorder, and risk of stroke. Neurology. (2008) 70:788–94. doi: 10.1212/01.wnl.0000304109.18563.81, PMID: 18316690

[56] Ohrnberger J Fichera E Sutton M. The dynamics of physical and mental health in the older population. J Econ Ageing. (2017) 9:52–62. doi: 10.1016/j.jeoa.2016.07.002, PMID: 28580276 PMC5446314

[57] Guo XJ Dash P Crawford JC Allen EK Zamora AE Boyd DF. Lung γδ T cells mediate protective responses during neonatal influenza infection that are associated with type 2 immunity. Immunity. (2018) 49:531–44.e6. doi: 10.1016/j.immuni.2018.07.011, PMID: 30170813 PMC6345262

[58] Palomino-Segura M Latino I Farsakoglu Y Gonzalez SF. Early production of IL-17A by γδ T cells in the trachea promotes viral clearance during influenza infection in mice. Eur J Immunol. (2020) 50:97–109. doi: 10.1002/eji.201948157, PMID: 31777067 PMC7003741

[59] Goldberg EL Molony RD Kudo E Sidorov S Kong Y Dixit VD. Ketogenic diet activates protective γδ T cell responses against influenza virus infection. Sci Immunol. (2019) 4:eaav2026. doi: 10.1126/sciimmunol.aav2026 31732517 PMC7189564

