# Supplementary material for: Healthy adults supplemented with a nutraceutical formulation containing Aloe vera gel, rosemary and Poria cocos enhances the effect of influenza vaccination in a randomized, triple-blind, placebo-controlled trial
Source: Front Nutr. 2023 Apr 24;10:1116634. doi: 10.3389/fnut.2023.1116634 (PMC10165552; doi:10.3389/fnut.2023.1116634)
Supplement: Supplementary file 1 [file Table_1.docx]

Supplementary Material

**Supplementary Table 1.** CONSORT 2010 checklist of information to include when reporting a randomised trial*

| Section/Topic | Item No | Checklist item | Reported on page No |
| --- | --- | --- | --- |
| Title and abstract | | | |
|  | 1a | Identification as a randomised trial in the title | 1 |
|  | 1b | Structured summary of trial design, methods, results, and conclusions (for specific guidance see CONSORT for abstracts) | 1 |
| Introduction | | | |
| Background and objectives | 2a | Scientific background and explanation of rationale | 2-3 |
|  | 2b | Specific objectives or hypotheses | 3 |
| Methods | | | |
| Trial design | 3a | Description of trial design (such as parallel, factorial) including allocation ratio | 3 |
|  | 3b | Important changes to methods after trial commencement (such as eligibility criteria), with reasons | N/A |
| Participants | 4a | Eligibility criteria for participants | 4 |
|  | 4b | Settings and locations where the data were collected | 3 |
| Interventions | 5 | The interventions for each group with sufficient details to allow replication, including how and when they were actually administered | 4-5 |
| Outcomes | 6a | Completely defined pre-specified primary and secondary outcome measures, including how and when they were assessed | 5-8 |
|  | 6b | Any changes to trial outcomes after the trial commenced, with reasons | N/A |
| Sample size | 7a | How sample size was determined | 8 |
|  | 7b | When applicable, explanation of any interim analyses and stopping guidelines | N/A |
| Randomisation: |  |  |  |
| Sequence generation | 8a | Method used to generate the random allocation sequence | 5 |
|  | 8b | Type of randomisation; details of any restriction (such as blocking and block size) | 5 |
| Allocation concealment mechanism | 9 | Mechanism used to implement the random allocation sequence (such as sequentially numbered containers), describing any steps taken to conceal the sequence until interventions were assigned | 5 |
| Implementation | 10 | Who generated the random allocation sequence, who enrolled participants, and who assigned participants to interventions | 5 |
| Blinding | 11a | If done, who was blinded after assignment to interventions (for example, participants, care providers, those assessing outcomes) and how | 5 |
|  | 11b | If relevant, description of the similarity of interventions | 5 |
| Statistical methods | 12a | Statistical methods used to compare groups for primary and secondary outcomes | 7-8 |
|  | 12b | Methods for additional analyses, such as subgroup analyses and adjusted analyses | 8-9 |
| Results | | | |
| Participant flow (a diagram is strongly recommended) | 13a | For each group, the numbers of participants who were randomly assigned, received intended treatment, and were analysed for the primary outcome | 9 |
|  | 13b | For each group, losses and exclusions after randomisation, together with reasons | 9 |
| Recruitment | 14a | Dates defining the periods of recruitment and follow-up | 3 |
|  | 14b | Why the trial ended or was stopped | N/A |
| Baseline data | 15 | A table showing baseline demographic and clinical characteristics for each group | 9, Table 1 |
| Numbers analysed | 16 | For each group, number of participants (denominator) included in each analysis and whether the analysis was by original assigned groups | 8-10, Table 2 – 3, Figures 1-2 |
| Outcomes and estimation | 17a | For each primary and secondary outcome, results for each group, and the estimated effect size and its precision (such as 95% confidence interval) | 9-11 Table 2 – 3, Figures 1-2 |
|  | 17b | For binary outcomes, presentation of both absolute and relative effect sizes is recommended | N/A |
| Ancillary analyses | 18 | Results of any other analyses performed, including subgroup analyses and adjusted analyses, distinguishing pre-specified from exploratory | N/A |
| Harms | 19 | All important harms or unintended effects in each group (for specific guidance see CONSORT for harms) | 11 |
| Discussion | | | |
| Limitations | 20 | Trial limitations, addressing sources of potential bias, imprecision, and, if relevant, multiplicity of analyses | 13 |
| Generalisability | 21 | Generalisability (external validity, applicability) of the trial findings | 13 |
| Interpretation | 22 | Interpretation consistent with results, balancing benefits and harms, and considering other relevant evidence | 11-14 |
| Other information | | |  |
| Registration | 23 | Registration number and name of trial registry | 2, 4 |
| Protocol | 24 | Where the full trial protocol can be accessed, if available | N/A |
| Funding | 25 | Sources of funding and other support (such as supply of drugs), role of funders | 14 |

*We strongly recommend reading this statement in conjunction with the CONSORT 2010 Explanation and Elaboration for important clarifications on all the items. If relevant, we also recommend reading CONSORT extensions for cluster randomised trials, non-inferiority and equivalence trials, non-pharmacological treatments, herbal interventions, and pragmatic trials. Additional extensions are forthcoming: for those and for up-to-date references relevant to this checklist, see [www.consort-statement.org](http://www.consort-statement.org).

**Supplementary Table 2.** Study population inclusion and exclusion criteria. Each participant was required to fulfill the inclusion criteria and not meet any of the exclusion criteria.

| **Inclusion Criteria** | **Exclusion Criteria** |
| --- | --- |
| Males and females between 40 and 80 years of age, inclusive | Women who are pregnant, breast feeding, or planning to become pregnant during the study |
| Female participant is not of child-bearing potential, defined as females who have undergone a sterilization procedure (e.g., hysterectomy, bilateral oophorectomy, bilateral tubal ligation, complete endometrial ablation) or have been post-menopausal for at least 1 year prior to screening | Participant has a known allergy to the active or inactive ingredients in UP360, UP446, placebo, or QIV |
| Females of child-bearing potential must have a negative baseline urine pregnancy test and agree to use a medically approved method of birth control for the duration of the study. All hormonal birth control must have been in use for a minimum of three months. | Unvaccinated participants with flu prior to baseline from September 2020 or prior to Day 28 vaccination |
| Healthy as determined by medical history and laboratory results as assessed by Medical Director | Participants self-reporting a diagnosis of COVID-19 prior to baseline or prior to Day 28 vaccination |
| Participants who have not yet but are willing to receive the influenza vaccine | Participants who have received the COVID-19 vaccine |
| Agrees to provide a verbal history of flu vaccination | Current use of prescribed immunomodulators (including corticosteroids) such as immunosuppressants or immunostimulants within 4 weeks of baseline |
| Agrees to maintain current lifestyle habits as much as possible throughout the study depending on your ability to maintain the following: diet, medications, supplements, exercise, and sleep and avoid taking new supplements | Current use of dietary supplement or herbal medicines associated with boosting or modulating the immune system unless willing to undergo a specified washout period prior to enrollment and agree not to take the supplements during the study  Examples: Senegalia catechu, Scutellaria baicalensis, Aloe vera, Rosemary extract, Echinacea, Astragalus, Ginseng, Turmeric, Medicinal mushroom, Probiotics, Beta-glucans |
| Willingness to complete questionnaires and diaries associated with the study and to complete all clinic visits | Participation in other clinical research studies 30 days prior to enrollment will be assessed on a case-by-case basis by the MD |
| Provided voluntary, written, informed consent to participate in the study | Individuals who are unable to give informed consent |
|  | Any other condition, chronic disease, or lifestyle factor, that, in the opinion of the MD, may adversely affect the participant's ability to complete the study or its measures or pose significant risk to the participant |


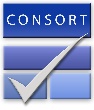
Supplementary Table 3. Upper respiratory tract infection (URTI) symptom severity, well and sick days from the Modified Wisconsin Upper Respiratory Symptom Survey (WURSS)-24 in the ITT population (n=50).

| **Variable** | **Study Timepoint** | **UP360**  **Mean ± SD**  **Median (Min to Max)**  **Within Group P-Value** | **Placebo**  **Mean ± SD**  **Median (Min to Max)**  **Within Group P-Value** | **UP360 vs. Placebo** |
| --- | --- | --- | --- | --- |
| Mean global severity index | Pre-Vaccination  (Baseline to Day 28) | 13.1 ± 30.9 0.0 (0.0 to 108.2) | 4.0 ± 13.6 0.0 (0.0 to 63.8) | 1.000 |
|  | Post-Vaccination  (Day 28 to Day 56) | 23.7 ± 60.6 0.0 (0.0 to 236.8) | 11.4 ± 36.5 0.0 (0.0 to 172.7) | 0.997 |
|  | Post-Vaccination vs. Pre-Vaccination | 11.8 ± 52.0 0.0 (-91.1 to 184.7) 0.998 | 7.2 ± 37.9 0.0 (-40.2 to 172.7) 1.000 | 1.000 |
| Mean symptom severity scores | Pre-Vaccination  (Baseline to Day 28) | 27.6 ± 33.1 19.5 (0.0 to 137.6) | 15.8 ± 21.3 0.0 (0.0 to 66.4) | 0.971 |
|  | Post-Vaccination  (Day 28 to Day 56) | 44.7 ± 76.9 0.5 (0.0 to 254.7) | 19.0 ± 27.8 0.0 (0.0 to 111.8) | 0.307 |
|  | Post-Vaccination vs. Pre-Vaccination | 17.0 ± 63.3 0.0 (-58.6 to 179.6) 0.700 | 2.5 ± 16.7 0.0 (-14.8 to 59.2) 1.000 | 0.975 |
| Number of well days | Pre-Vaccination  (Baseline to Day 28) | 27.2 ± 1.7 28.0 (21.3 to 28.0) | 27.9 ± 0.3 28.0 (26.9 to 28.0) | 0.981 |
|  | Post-Vaccination  (Day 28 to Day 56) | 27.3 ± 1.8 28.0 (21.0 to 28.0) | 27.6 ± 1.3 28.0 (23.0 to 28.0) | 1.000 |
|  | Post-Vaccination vs. Pre-Vaccination | -0.2 ± 2.2 0.0 (-7.0 to 4.1) 1.000 | -0.3 ± 1.4 0.0 (-5.0 to 1.1) 0.984 | 1.000 |
| Number of sick days | Pre-Vaccination  (Baseline to Day 28) | 0.8 ± 1.7 0.0 (0.0 to 6.7) | 0.1 ± 0.3 0.0 (0.0 to 1.1) | 0.855 |
|  | Post-Vaccination  (Day 28 to Day 56) | 0.7 ± 1.8 0.0 (0.0 to 7.0) | 0.4 ± 1.3 0.0 (0.0 to 5.0) | 0.999 |
|  | Post-Vaccination vs. Pre-Vaccination | 0.2 ± 2.2 0.0 (-4.1 to 7.0) 1.000 | 0.3 ± 1.4 0.0 (-1.1 to 5.0) 0.984 | 1.000 |


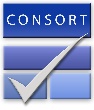
Supplementary Table 4. Vitality and Quality of Life Questionnaire in the ITT population (n=50)

| **Question** | **Description** | **Study Timepoint** | **UP360**  **Mean ± SD**  **Median (Min to Max)**  **Within Group P-Value** | **Placebo**  **Mean ± SD**  **Median (Min to Max)**  **Within Group P-Value** | **UP360 vs. Placebo** |
| --- | --- | --- | --- | --- | --- |
| 1 | I feel alive and vital | Baseline | 5.7 ± 0.9 6.0 (4.0 to 7.0) | 5.7 ± 1.1 6.0 (3.0 to 7.0) | 1.000 |
|  |  | Day 28 | 5.6 ± 1.0 6.0 (3.0 to 7.0) | 5.4 ± 1.6 6.0 (1.0 to 7.0) | 0.999 |
|  |  | Day 56 | 5.6 ± 0.9 6.0 (4.0 to 7.0) | 5.8 ± 1.2 6.0 (3.0 to 7.0) | 1.000 |
|  |  | Pre-Vaccination  (Baseline to Day 28) | -0.1 ± 1.1 0.0 (-3.0 to 2.0) 1.000 | -0.3 ± 1.7 0.0 (-6.0 to 2.0) 0.862 | 0.981 |
|  |  | Baseline to Day 56 | -0.1 ± 0.8 0.0 (-2.0 to 1.0) 1.000 | 0.0 ± 0.8 0.0 (-1.0 to 2.0) 1.000 | 1.000 |
|  |  | Post-Vaccination  (Day 28 to Day 56) | 0.0 ± 0.6 0.0 (-1.0 to 1.0) 1.000 | 0.3 ± 1.6 0.0 (-2.0 to 6.0) 0.833 | 1.000 |
| 2 | I don't feel very energetic | Baseline | 3.3 ± 1.2 3.0 (1.0 to 5.0) | 3.3 ± 1.2 4.0 (1.0 to 5.0) | 1.000 |
|  |  | Day 28 | 3.5 ± 1.2 4.0 (1.0 to 5.0) | 3.0 ± 1.3 2.5 (1.0 to 6.0) | 0.943 |
|  |  | Day 56 | 3.2 ± 1.3 3.0 (1.0 to 6.0) | 3.1 ± 1.2 3.0 (1.0 to 5.0) | 1.000 |
|  |  | Pre-Vaccination  (Baseline to Day 28) | 0.0 ± 1.1 0.0 (-3.0 to 2.0) 1.000 | -0.3 ± 1.5 0.0 (-3.0 to 3.0) 0.980 | 0.994 |
|  |  | Baseline to Day 56 | -0.2 ± 1.4 0.0 (-4.0 to 2.0) 1.000 | -0.2 ± 1.2 0.0 (-3.0 to 2.0) 1.000 | 1.000 |
|  |  | Post-Vaccination  (Day 28 to Day 56) | -0.3 ± 1.4 0.0 (-3.0 to 2.0) 0.996 | 0.2 ± 1.4 0.0 (-3.0 to 3.0) 1.000 | 0.996 |
| 3 | Sometimes I feel so alive I just want to burst | Baseline | 3.3 ± 1.5 3.0 (1.0 to 6.0) | 3.8 ± 1.6 4.0 (1.0 to 6.0) | 0.963 |
|  |  | Day 28 | 3.5 ± 1.5 3.0 (1.0 to 7.0) | 3.9 ± 1.6 4.0 (1.0 to 7.0) | 0.999 |
|  |  | Day 56 | 3.3 ± 1.4 3.0 (1.0 to 6.0) | 3.7 ± 1.6 4.0 (1.0 to 6.0) | 1.000 |
|  |  | Pre-Vaccination  (Baseline to Day 28) | 0.3 ± 1.4 0.0 (-2.0 to 4.0) 0.978 | 0.0 ± 1.3 0.0 (-2.0 to 2.0) 1.000 | 0.927 |
|  |  | Baseline to Day 56 | 0.2 ± 1.3 0.0 (-1.0 to 4.0) 1.000 | -0.2 ± 0.9 0.0 (-2.0 to 1.0) 1.000 | 0.995 |
|  |  | Post-Vaccination  (Day 28 to Day 56) | -0.2 ± 1.1 0.0 (-3.0 to 2.0) 1.000 | -0.2 ± 1.4 0.0 (-4.0 to 2.0) 0.998 | 1.000 |
| 4 | I have energy and spirit | Baseline | 5.2 ± 1.0 5.0 (4.0 to 7.0) | 5.3 ± 1.2 6.0 (2.0 to 7.0) | 1.000 |
|  |  | Day 28 | 5.0 ± 1.1 5.0 (2.0 to 7.0) | 5.5 ± 1.2 6.0 (2.0 to 7.0) | 0.975 |
|  |  | Day 56 | 5.2 ± 1.1 5.0 (3.0 to 7.0) | 5.5 ± 1.2 6.0 (3.0 to 7.0) | 0.999 |
|  |  | Pre-Vaccination  (Baseline to Day 28) | -0.1 ± 0.8 0.0 (-2.0 to 1.0) 1.000 | 0.1 ± 1.1 0.0 (-1.0 to 4.0) 0.999 | 0.998 |
|  |  | Baseline to Day 56 | 0.0 ± 0.8 0.0 (-2.0 to 2.0) 1.000 | 0.1 ± 1.0 0.0 (-2.0 to 3.0) 0.999 | 0.998 |
|  |  | Post-Vaccination  (Day 28 to Day 56) | 0.1 ± 0.7 0.0 (-1.0 to 2.0) 0.999 | 0.0 ± 0.8 0.0 (-2.0 to 2.0) 1.000 | 0.975 |
| 5 | I look forward to each new day | Baseline | 5.8 ± 0.9 6.0 (4.0 to 7.0) | 5.8 ± 1.3 6.0 (2.0 to 7.0) | 1.000 |
|  |  | Day 28 | 5.6 ± 1.2 6.0 (3.0 to 7.0) | 6.0 ± 1.4 6.0 (2.0 to 7.0) | 0.996 |
|  |  | Day 56 | 5.6 ± 1.2 5.0 (3.0 to 7.0) | 5.8 ± 1.2 6.0 (3.0 to 7.0) | 1.000 |
|  |  | Pre-Vaccination  (Baseline to Day 28) | -0.1 ± 0.8 0.0 (-2.0 to 1.0) 0.990 | 0.1 ± 0.7 0.0 (-1.0 to 2.0) 0.991 | 1.000 |
|  |  | Baseline to Day 56 | -0.1 ± 0.8 0.0 (-2.0 to 1.0) 0.990 | -0.0 ± 0.6 0.0 (-1.0 to 1.0) 1.000 | 1.000 |
|  |  | Post-Vaccination  (Day 28 to Day 56) | 0.0 ± 0.7 0.0 (-2.0 to 1.0) 1.000 | -0.2 ± 0.6 0.0 (-1.0 to 1.0) 0.971 | 1.000 |
| 6 | I always feel alert and awake | Baseline | 5.0 ± 1.2 5.0 (2.0 to 7.0) | 5.1 ± 1.1 5.0 (2.0 to 6.0) | 1.000 |
|  |  | Day 28 | 4.8 ± 1.3 5.0 (1.0 to 6.0) | 5.0 ± 1.3 5.0 (1.0 to 7.0) | 1.000 |
|  |  | Day 56 | 4.8 ± 1.2 5.0 (3.0 to 7.0) | 5.5 ± 1.0 6.0 (3.0 to 7.0) | 0.766 |
|  |  | Pre-Vaccination  (Baseline to Day 28) | -0.0 ± 1.4 0.0 (-3.0 to 3.0) 1.000 | -0.1 ± 1.4 0.0 (-5.0 to 2.0) 1.000 | 1.000 |
|  |  | Baseline to Day 56 | -0.0 ± 1.1 0.0 (-1.0 to 2.0) 1.000 | 0.3 ± 0.8 0.0 (-1.0 to 2.0) 0.921 | 0.913 |
|  |  | Post-Vaccination  (Day 28 to Day 56) | 0.0 ± 1.0 0.0 (-2.0 to 2.0) 1.000 | 0.4 ± 1.4 0.0 (-1.0 to 6.0) 0.839 | 0.766 |
| 7 | I feel energized when I wake up | Baseline | 4.6 ± 1.5 5.0 (1.0 to 7.0) | 4.9 ± 1.3 5.0 (1.0 to 7.0) | 0.999 |
|  |  | Day 28 | 4.5 ± 1.7 5.0 (1.0 to 7.0) | 5.2 ± 1.4 6.0 (2.0 to 7.0) | 0.804 |
|  |  | Day 56 | 4.4 ± 1.5 4.0 (1.0 to 7.0) | 5.2 ± 1.4 5.5 (2.0 to 7.0) | 0.739 |
|  |  | Pre-Vaccination  (Baseline to Day 28) | -0.1 ± 1.3 0.0 (-3.0 to 3.0) 0.999 | 0.2 ± 1.1 0.0 (-3.0 to 2.0) 0.972 | 0.938 |
|  |  | Baseline to Day 56 | -0.2 ± 1.1 0.0 (-2.0 to 3.0) 0.977 | 0.2 ± 1.0 0.0 (-2.0 to 2.0) 0.992 | 0.962 |
|  |  | Post-Vaccination  (Day 28 to Day 56) | -0.1 ± 0.7 0.0 (-1.0 to 1.0) 1.000 | -0.0 ± 0.6 0.0 (-1.0 to 1.0) 1.000 | 0.855 |
| 8 | I feel energy and vitality throughout the day | Baseline | 5.2 ± 0.9 5.0 (4.0 to 7.0) | 5.0 ± 1.3 6.0 (2.0 to 6.0) | 1.000 |
|  |  | Day 28 | 5.1 ± 0.9 5.0 (3.0 to 7.0) | 5.1 ± 1.1 5.0 (2.0 to 7.0) | 1.000 |
|  |  | Day 56 | 5.0 ± 1.0 5.0 (3.0 to 7.0) | 5.3 ± 1.0 5.5 (3.0 to 7.0) | 0.998 |
|  |  | Pre-Vaccination  (Baseline to Day 28) | 0.0 ± 1.0 0.0 (-2.0 to 2.0) 1.000 | 0.0 ± 1.0 0.0 (-1.0 to 3.0) 1.000 | 1.000 |
|  |  | Baseline to Day 56 | -0.1 ± 1.0 0.0 (-2.0 to 2.0) 0.998 | 0.2 ± 1.0 0.0 (-1.0 to 3.0) 0.979 | 1.000 |
|  |  | Post-Vaccination  (Day 28 to Day 56) | -0.1 ± 0.7 0.0 (-2.0 to 1.0) 0.999 | 0.2 ± 0.8 0.0 (-1.0 to 2.0) 0.993 | 1.000 |
| 9 | I have a midday slump in energy | Baseline | 3.9 ± 1.6 4.0 (1.0 to 7.0) | 4.1 ± 1.3 4.0 (2.0 to 7.0) | 1.000 |
|  |  | Day 28 | 4.1 ± 1.1 4.0 (2.0 to 6.0) | 3.7 ± 1.4 4.0 (1.0 to 6.0) | 0.995 |
|  |  | Day 56 | 4.1 ± 1.2 4.0 (1.0 to 6.0) | 3.9 ± 1.1 4.0 (1.0 to 6.0) | 1.000 |
|  |  | Pre-Vaccination  (Baseline to Day 28) | 0.0 ± 1.1 0.0 (-2.0 to 3.0) 1.000 | -0.3 ± 1.0 0.0 (-3.0 to 1.0) 0.905 | 1.000 |
|  |  | Baseline to Day 56 | 0.0 ± 1.7 0.0 (-3.0 to 5.0) 1.000 | -0.1 ± 1.1 0.0 (-3.0 to 2.0) 1.000 | 1.000 |
|  |  | Post-Vaccination  (Day 28 to Day 56) | -0.0 ± 1.1 0.0 (-3.0 to 2.0) 1.000 | 0.2 ± 1.1 0.0 (-1.0 to 2.0) 0.992 | 1.000 |
| 10 | I feel engaged and enthusiastic in my personal relationships (friends and family) | Baseline | 5.6 ± 1.4 6.0 (2.0 to 7.0) | 5.7 ± 1.2 6.0 (2.0 to 7.0) | 1.000 |
|  |  | Day 28 | 5.6 ± 1.1 6.0 (3.0 to 7.0) | 5.5 ± 1.3 6.0 (2.0 to 7.0) | 0.995 |
|  |  | Day 56 | 5.5 ± 1.2 6.0 (2.0 to 7.0) | 5.7 ± 1.0 6.0 (3.0 to 7.0) | 1.000 |
|  |  | Pre-Vaccination  (Baseline to Day 28) | 0.1 ± 1.2 0.0 (-4.0 to 2.0) 1.000 | -0.3 ± 1.0 0.0 (-2.0 to 2.0) 0.836 | 0.999 |
|  |  | Baseline to Day 56 | -0.0 ± 0.8 0.0 (-1.0 to 2.0) 1.000 | -0.1 ± 1.0 0.0 (-2.0 to 2.0) 1.000 | 1.000 |
|  |  | Post-Vaccination  (Day 28 to Day 56) | -0.1 ± 1.0 0.0 (-2.0 to 3.0) 0.999 | 0.2 ± 1.0 0.0 (-2.0 to 3.0) 0.930 | 1.000 |
| 11 | I have good mental clarity and focus | Baseline | 5.6 ± 0.9 5.0 (4.0 to 7.0) | 5.6 ± 1.1 6.0 (2.0 to 7.0) | 1.000 |
|  |  | Day 28 | 5.6 ± 0.8 5.0 (4.0 to 7.0) | 5.8 ± 1.0 6.0 (2.0 to 7.0) | 1.000 |
|  |  | Day 56 | 5.4 ± 0.8 5.0 (4.0 to 7.0) | 5.7 ± 0.8 6.0 (4.0 to 7.0) | 0.999 |
|  |  | Pre-Vaccination  (Baseline to Day 28) | 0.2 ± 0.7 0.0 (-1.0 to 2.0) 0.996 | 0.1 ± 0.7 0.0 (-1.0 to 2.0) 0.997 | 1.000 |
|  |  | Baseline to Day 56 | -0.0 ± 0.8 0.0 (-1.0 to 2.0) 1.000 | 0.0 ± 0.9 0.0 (-2.0 to 2.0) 1.000 | 1.000 |
|  |  | Post-Vaccination  (Day 28 to Day 56) | -0.2 ± 0.6 0.0 (-1.0 to 1.0) 0.904 | -0.0 ± 0.7 0.0 (-1.0 to 2.0) 1.000 | 1.000 |
| 12 | I feel I have a good sense of purpose and meaning in my life | Baseline | 5.9 ± 0.8 6.0 (5.0 to 7.0) | 5.8 ± 1.2 6.0 (2.0 to 7.0) | 1.000 |
|  |  | Day 28 | 5.8 ± 1.1 6.0 (3.0 to 7.0) | 5.6 ± 1.3 6.0 (2.0 to 7.0) | 0.990 |
|  |  | Day 56 | 5.7 ± 0.9 6.0 (4.0 to 7.0) | 5.6 ± 1.3 6.0 (2.0 to 7.0) | 0.999 |
|  |  | Pre-Vaccination  (Baseline to Day 28) | -0.1 ± 0.8 0.0 (-2.0 to 1.0) 0.999 | -0.3 ± 0.6 0.0 (-2.0 to 1.0) 0.610 | 0.938 |
|  |  | Baseline to Day 56 | -0.2 ± 0.6 0.0 (-1.0 to 1.0) 0.808 | -0.3 ± 0.7 0.0 (-2.0 to 1.0) 0.409 | 0.894 |
|  |  | Post-Vaccination  (Day 28 to Day 56) | -0.1 ± 0.6 0.0 (-1.0 to 1.0) 0.992 | -0.0 ± 0.6 0.0 (-1.0 to 1.0) 1.000 | 0.976 |
| 13 | It takes a great effort to start things. This applies to everyday activities such as getting out of bed, washing myself, and eating | Baseline | 2.6 ± 1.4 2.0 (1.0 to 7.0) | 2.4 ± 1.2 2.0 (1.0 to 5.0) | 1.000 |
|  |  | Day 28 | 2.9 ± 1.8 2.0 (1.0 to 7.0) | 2.0 ± 1.1 2.0 (1.0 to 5.0) | 0.243 |
|  |  | Day 56 | 2.6 ± 1.2 2.0 (1.0 to 4.0) | 2.2 ± 1.1 2.0 (1.0 to 5.0) | 0.988 |
|  |  | Pre-Vaccination  (Baseline to Day 28) | 0.5 ± 1.6 0.0 (-1.0 to 5.0) 0.916 | -0.4 ± 1.3 0.0 (-3.0 to 3.0) 0.878 | 0.873 |
|  |  | Baseline to Day 56 | 0.2 ± 1.0 0.0 (-2.0 to 2.0) 1.000 | -0.1 ± 1.4 0.0 (-3.0 to 3.0) 0.999 | 0.997 |
|  |  | Post-Vaccination  (Day 28 to Day 56) | -0.3 ± 1.9 0.0 (-6.0 to 2.0) 0.972 | 0.2 ± 0.8 0.0 (-1.0 to 2.0) 0.996 | 0.645 |
| 14 | I forget things slightly more often than I should, but I am able to manage by making notes | Baseline | 3.2 ± 1.5 3.0 (1.0 to 6.0) | 3.5 ± 1.5 4.0 (1.0 to 6.0) | 0.998 |
|  |  | Day 28 | 3.7 ± 1.7 4.0 (1.0 to 7.0) | 3.0 ± 1.5 3.0 (1.0 to 6.0) | 0.961 |
|  |  | Day 56 | 3.6 ± 1.4 4.0 (2.0 to 7.0) | 3.2 ± 1.5 3.0 (1.0 to 6.0) | 1.000 |
|  |  | Pre-Vaccination  (Baseline to Day 28) | 0.4 ± 1.0 0.0 (-1.0 to 3.0) 0.585 | -0.3 ± 1.5 0.0 (-3.0 to 3.0) 0.848 | 1.000 |
|  |  | Baseline to Day 56 | 0.3 ± 1.2 0.0 (-3.0 to 3.0) 0.891 | -0.1 ± 1.3 0.0 (-4.0 to 2.0) 0.999 | 1.000 |
|  |  | Post-Vaccination  (Day 28 to Day 56) | -0.1 ± 1.3 0.0 (-4.0 to 1.0) 1.000 | 0.2 ± 1.0 0.0 (-3.0 to 2.0) 0.994 | 0.999 |
| 15 | My thoughts are neither slow nor sluggish when it comes to work involving mental effort | Baseline | 4.8 ± 1.5 5.0 (2.0 to 7.0) | 4.0 ± 1.8 4.0 (1.0 to 6.0) | 0.795 |
|  |  | Day 28 | 4.6 ± 1.5 5.0 (2.0 to 7.0) | 3.8 ± 1.7 4.0 (1.0 to 6.0) | 0.693 |
|  |  | Day 56 | 4.6 ± 1.4 5.0 (2.0 to 7.0) | 4.4 ± 1.6 5.0 (1.0 to 6.0) | 1.000 |
|  |  | Pre-Vaccination  (Baseline to Day 28) | 0.0 ± 1.7 0.0 (-4.0 to 4.0) 1.000 | -0.3 ± 2.9 0.0 (-5.0 to 5.0) 1.000 | 0.507 |
|  |  | Baseline to Day 56 | -0.0 ± 1.7 0.0 (-4.0 to 3.0) 1.000 | 0.4 ± 2.1 0.0 (-5.0 to 5.0) 0.986 | 0.998 |
|  |  | Post-Vaccination  (Day 28 to Day 56) | -0.0 ± 1.9 0.0 (-5.0 to 3.0) 1.000 | 0.7 ± 1.9 0.0 (-4.0 to 5.0) 0.857 | 1.000 |
| 16 | My thoughts often feel slow and sluggish, even when carrying out everyday activities, for example, a conversation with a person or when reading the newspaper. | Baseline | 2.3 ± 1.1 2.0 (1.0 to 5.0) | 2.1 ± 1.1 2.0 (1.0 to 5.0) | 0.999 |
|  |  | Day 28 | 2.3 ± 1.1 2.0 (1.0 to 5.0) | 2.1 ± 1.1 2.0 (1.0 to 5.0) | 1.000 |
|  |  | Day 56 | 2.2 ± 0.8 2.0 (1.0 to 4.0) | 2.3 ± 1.1 2.0 (1.0 to 6.0) | 0.993 |
|  |  | Pre-Vaccination  (Baseline to Day 28) | -0.1 ± 1.2 0.0 (-3.0 to 3.0) 1.000 | 0.1 ± 0.8 0.0 (-1.0 to 2.0) 1.000 | 1.000 |
|  |  | Baseline to Day 56 | -0.2 ± 1.2 0.0 (-3.0 to 2.0) 0.993 | 0.3 ± 0.9 0.0 (-1.0 to 3.0) 0.904 | 1.000 |
|  |  | Post-Vaccination  (Day 28 to Day 56) | -0.1 ± 0.9 0.0 (-3.0 to 1.0) 0.999 | 0.2 ± 1.2 0.0 (-3.0 to 2.0) 0.946 | 1.000 |
| 17 | I become stressed easily | Baseline | 2.9 ± 1.2 3.0 (1.0 to 5.0) | 2.8 ± 1.3 2.0 (1.0 to 6.0) | 1.000 |
|  |  | Day 28 | 3.5 ± 1.3 4.0 (2.0 to 6.0) | 2.7 ± 1.1 3.0 (1.0 to 5.0) | 0.744 |
|  |  | Day 56 | 3.2 ± 1.1 3.0 (1.0 to 6.0) | 2.8 ± 1.1 2.5 (1.0 to 4.0) | 0.998 |
|  |  | Pre-Vaccination  (Baseline to Day 28) | 0.4 ± 1.4 0.0 (-1.0 to 4.0) 0.368 | 0.0 ± 0.7 0.0 (-1.0 to 1.0) 1.000 | 1.000 |
|  |  | Baseline to Day 56 | 0.1 ± 0.8 0.0 (-1.0 to 2.0) 0.996 | 0.0 ± 1.2 0.0 (-4.0 to 2.0) 1.000 | 1.000 |
|  |  | Post-Vaccination  (Day 28 to Day 56) | -0.3 ± 1.1 0.0 (-3.0 to 2.0) 0.876 | 0.0 ± 1.0 0.0 (-3.0 to 2.0) 1.000 | 0.810 |
| 18 | I am often short-tempered or irritable | Baseline | 2.5 ± 1.2 2.0 (1.0 to 5.0) | 2.5 ± 1.2 2.0 (1.0 to 5.0) | 1.000 |
|  |  | Day 28 | 2.4 ± 0.8 2.0 (1.0 to 4.0) | 2.2 ± 1.1 2.0 (1.0 to 4.0) | 1.000 |
|  |  | Day 56 | 2.4 ± 1.0 2.0 (1.0 to 4.0) | 2.2 ± 1.2 2.0 (1.0 to 5.0) | 1.000 |
|  |  | Pre-Vaccination  (Baseline to Day 28) | -0.1 ± 0.7 0.0 (-2.0 to 1.0) 1.000 | -0.2 ± 0.7 0.0 (-1.0 to 1.0) 0.959 | 1.000 |
|  |  | Baseline to Day 56 | -0.2 ± 0.8 0.0 (-2.0 to 1.0) 0.998 | -0.2 ± 1.1 0.0 (-3.0 to 2.0) 0.959 | 1.000 |
|  |  | Post-Vaccination  (Day 28 to Day 56) | -0.0 ± 0.8 0.0 (-2.0 to 1.0) 1.000 | 0.0 ± 0.9 0.0 (-2.0 to 2.0) 1.000 | 1.000 |
| 19 | I become irritated very quickly about small things or things that do not bother other people | Baseline | 2.5 ± 1.4 2.0 (1.0 to 6.0) | 2.1 ± 1.0 2.0 (1.0 to 5.0) | 0.946 |
|  |  | Day 28 | 2.3 ± 0.9 2.0 (1.0 to 4.0) | 2.1 ± 0.9 2.0 (1.0 to 4.0) | 1.000 |
|  |  | Day 56 | 2.4 ± 0.9 2.0 (1.0 to 4.0) | 2.2 ± 1.0 2.0 (1.0 to 4.0) | 1.000 |
|  |  | Pre-Vaccination  (Baseline to Day 28) | -0.3 ± 1.3 0.0 (-4.0 to 2.0) 0.985 | 0.0 ± 0.8 0.0 (-1.0 to 2.0) 1.000 | 0.936 |
|  |  | Baseline to Day 56 | -0.2 ± 1.0 0.0 (-2.0 to 2.0) 1.000 | 0.2 ± 1.0 0.0 (-3.0 to 2.0) 0.999 | 0.998 |
|  |  | Post-Vaccination  (Day 28 to Day 56) | 0.1 ± 1.1 0.0 (-2.0 to 2.0) 1.000 | 0.2 ± 1.1 0.0 (-3.0 to 2.0) 0.998 | 1.000 |
| 20 | I do not get enough sleep | Baseline | 3.8 ± 1.7 4.0 (1.0 to 7.0) | 3.7 ± 1.3 4.0 (2.0 to 7.0) | 1.000 |
|  |  | Day 28 | 3.4 ± 1.6 4.0 (1.0 to 7.0) | 3.1 ± 1.5 3.0 (1.0 to 6.0) | 1.000 |
|  |  | Day 56 | 3.7 ± 1.7 3.0 (1.0 to 7.0) | 3.5 ± 1.7 3.5 (1.0 to 7.0) | 1.000 |
|  |  | Pre-Vaccination  (Baseline to Day 28) | -0.5 ± 1.8 0.0 (-6.0 to 2.0) 0.817 | -0.5 ± 1.6 0.0 (-3.0 to 2.0) 0.568 | 0.839 |
|  |  | Baseline to Day 56 | -0.3 ± 1.7 0.0 (-6.0 to 2.0) 0.996 | -0.2 ± 1.4 0.0 (-3.0 to 3.0) 0.999 | 0.999 |
|  |  | Post-Vaccination  (Day 28 to Day 56) | 0.2 ± 0.9 0.0 (-1.0 to 2.0) 0.998 | 0.4 ± 1.4 0.0 (-2.0 to 6.0) 0.929 | 1.000 |
| 21 | I have slight problems falling asleep or my sleep is shorter, lighter, or more restless | Baseline | 3.4 ± 1.7 4.0 (1.0 to 6.0) | 3.4 ± 1.8 3.0 (1.0 to 7.0) | 1.000 |
|  |  | Day 28 | 3.7 ± 1.7 4.0 (1.0 to 7.0) | 2.8 ± 1.5 2.0 (1.0 to 6.0) | 0.873 |
|  |  | Day 56 | 3.9 ± 1.9 3.0 (2.0 to 7.0) | 3.3 ± 1.8 3.0 (1.0 to 6.0) | 0.994 |
|  |  | Pre-Vaccination  (Baseline to Day 28) | 0.1 ± 1.2 0.0 (-2.0 to 3.0) 1.000 | -0.6 ± 1.6 -0.5 (-5.0 to 2.0) 0.358 | 0.955 |
|  |  | Baseline to Day 56 | 0.3 ± 1.5 0.0 (-4.0 to 3.0) 0.914 | -0.1 ± 1.7 0.0 (-5.0 to 3.0) 1.000 | 1.000 |
|  |  | Post-Vaccination  (Day 28 to Day 56) | 0.2 ± 1.0 0.0 (-2.0 to 2.0) 0.995 | 0.5 ± 0.7 0.0 (-1.0 to 2.0) 0.567 | 1.000 |
| 22 | My energy level fluctuates throughout the day. I can predict that I will feel better at certain times and worse at other times. | Baseline | 3.6 ± 1.3 4.0 (1.0 to 6.0) | 3.2 ± 1.3 3.0 (1.0 to 6.0) | 0.964 |
|  |  | Day 28 | 3.7 ± 1.4 4.0 (1.0 to 7.0) | 3.0 ± 1.4 3.0 (1.0 to 6.0) | 0.832 |
|  |  | Day 56 | 3.9 ± 1.1 4.0 (2.0 to 6.0) | 3.3 ± 1.4 3.5 (1.0 to 6.0) | 0.968 |
|  |  | Baseline to Day 28 | -0.0 ± 1.3 0.0 (-3.0 to 3.0) 1.000 | -0.1 ± 1.5 0.0 (-3.0 to 3.0) 1.000 | 0.891 |
|  |  | Baseline to Day 56 | 0.1 ± 0.9 0.0 (-2.0 to 2.0) 0.999 | 0.2 ± 1.2 0.0 (-3.0 to 2.0) 0.999 | 0.999 |
|  |  | Post-Vaccination  (Day 28 to Day 56) | 0.1 ± 1.2 0.0 (-2.0 to 3.0) 1.000 | 0.3 ± 1.3 0.0 (-3.0 to 4.0) 0.986 | 0.997 |
| 23 | I feel unwell at all times of the day and night | Baseline | 1.4 ± 0.7 1.0 (1.0 to 4.0) | 1.5 ± 0.8 1.0 (1.0 to 4.0) | 0.999 |
|  |  | Day 28 | 1.3 ± 0.4 1.0 (1.0 to 2.0) | 1.5 ± 0.7 1.0 (1.0 to 3.0) | 0.988 |
|  |  | Day 56 | 1.4 ± 0.7 1.0 (1.0 to 4.0) | 1.9 ± 1.2 1.0 (1.0 to 4.0) | 0.569 |
|  |  | Pre-Vaccination  (Baseline to Day 28) | -0.1 ± 0.6 0.0 (-2.0 to 1.0) 1.000 | -0.0 ± 0.9 0.0 (-2.0 to 2.0) 1.000 | 1.000 |
|  |  | Baseline to Day 56 | 0.0 ± 1.0 0.0 (-2.0 to 3.0) 1.000 | 0.3 ± 1.0 0.0 (-1.0 to 3.0) 0.766 | 0.476 |
|  |  | Post-Vaccination  (Day 28 to Day 56) | 0.1 ± 0.8 0.0 (-1.0 to 3.0) 1.000 | 0.4 ± 1.0 0.0 (-1.0 to 3.0) 0.698 | 0.242 |
| 24 | I wake up feeling tired | Baseline | 3.1 ± 1.6 3.0 (1.0 to 7.0) | 3.3 ± 1.5 3.0 (1.0 to 7.0) | 1.000 |
|  |  | Day 28 | 3.4 ± 1.6 4.0 (1.0 to 6.0) | 2.8 ± 1.5 2.5 (1.0 to 6.0) | 0.981 |
|  |  | Day 56 | 3.3 ± 1.5 3.0 (1.0 to 7.0) | 2.8 ± 1.5 2.0 (1.0 to 6.0) | 0.998 |
|  |  | Pre-Vaccination  (Baseline to Day 28) | 0.2 ± 1.1 0.0 (-2.0 to 2.0) 0.991 | -0.4 ± 1.2 0.0 (-3.0 to 2.0) 0.713 | 1.000 |
|  |  | Baseline to Day 56 | 0.0 ± 1.2 0.0 (-3.0 to 2.0) 1.000 | -0.4 ± 1.3 0.0 (-3.0 to 2.0) 0.713 | 1.000 |
|  |  | Post-Vaccination  (Day 28 to Day 56) | -0.1 ± 0.9 0.0 (-2.0 to 2.0) 1.000 | 0.0 ± 0.5 0.0 (-1.0 to 1.0) 1.000 | 0.981 |
| 25 | I have a hard time participating in vigorous activity | Baseline | 2.7 ± 1.3 2.0 (1.0 to 7.0) | 2.4 ± 1.3 2.0 (1.0 to 5.0) | 0.998 |
|  |  | Day 28 | 2.8 ± 1.4 2.0 (1.0 to 7.0) | 2.2 ± 1.1 2.0 (1.0 to 5.0) | 0.964 |
|  |  | Day 56 | 2.8 ± 1.3 2.0 (1.0 to 7.0) | 2.7 ± 1.3 2.0 (1.0 to 5.0) | 1.000 |
|  |  | Pre-Vaccination  (Baseline to Day 28) | 0.0 ± 0.6 0.0 (-1.0 to 2.0) 1.000 | -0.1 ± 0.7 0.0 (-2.0 to 1.0) 1.000 | 0.987 |
|  |  | Baseline to Day 56 | 0.0 ± 0.7 0.0 (-1.0 to 1.0) 1.000 | 0.3 ± 1.1 0.0 (-3.0 to 3.0) 0.826 | 1.000 |
|  |  | Post-Vaccination  (Day 28 to Day 56) | -0.0 ± 0.6 0.0 (-2.0 to 1.0) 1.000 | 0.4 ± 0.9 0.0 (-1.0 to 3.0) 0.512 | 1.000 |
| 26 | I don't do much during the day | Baseline | 2.4 ± 1.3 2.0 (1.0 to 6.0) | 2.5 ± 1.4 2.0 (1.0 to 5.0) | 1.000 |
|  |  | Day 28 | 2.5 ± 1.5 2.0 (1.0 to 6.0) | 2.3 ± 1.2 2.0 (1.0 to 5.0) | 1.000 |
|  |  | Day 56 | 2.7 ± 1.3 2.0 (1.0 to 6.0) | 2.6 ± 1.3 2.0 (1.0 to 6.0) | 1.000 |
|  |  | Pre-Vaccination  (Baseline to Day 28) | 0.0 ± 0.9 0.0 (-1.0 to 2.0) 1.000 | -0.2 ± 1.1 0.0 (-3.0 to 1.0) 0.987 | 1.000 |
|  |  | Baseline to Day 56 | 0.3 ± 0.7 0.0 (-1.0 to 2.0) 0.961 | 0.0 ± 1.0 0.0 (-2.0 to 2.0) 1.000 | 1.000 |
|  |  | Post-Vaccination  (Day 28 to Day 56) | 0.3 ± 0.6 0.0 (-1.0 to 1.0) 0.973 | 0.3 ± 1.1 0.0 (-2.0 to 4.0) 0.941 | 1.000 |
| 27 | I have enough energy for everyday life | Baseline | 5.7 ± 1.1 6.0 (3.0 to 7.0) | 5.9 ± 1.6 7.0 (1.0 to 7.0) | 1.000 |
|  |  | Day 28 | 5.7 ± 0.8 6.0 (4.0 to 7.0) | 5.6 ± 1.6 6.0 (2.0 to 7.0) | 1.000 |
|  |  | Day 56 | 5.3 ± 1.2 6.0 (2.0 to 7.0) | 5.4 ± 1.7 6.0 (1.0 to 7.0) | 1.000 |
|  |  | Pre-Vaccination  (Baseline to Day 28) | 0.0 ± 0.8 0.0 (-1.0 to 2.0) 1.000 | -0.3 ± 1.0 0.0 (-3.0 to 2.0) 0.894 | 1.000 |
|  |  | Baseline to Day 56 | -0.3 ± 1.1 0.0 (-3.0 to 3.0) 0.879 | -0.5 ± 1.5 0.0 (-5.0 to 3.0) 0.333 | 0.991 |
|  |  | Post-Vaccination  (Day 28 to Day 56) | -0.3 ± 1.1 0.0 (-4.0 to 1.0) 0.853 | -0.2 ± 1.6 0.0 (-5.0 to 3.0) 0.992 | 0.989 |
| 28 | I have problems starting things | Baseline | 2.6 ± 1.5 2.0 (1.0 to 7.0) | 2.3 ± 1.3 2.0 (1.0 to 5.0) | 0.996 |
|  |  | Day 28 | 2.8 ± 1.3 2.0 (1.0 to 6.0) | 2.4 ± 1.1 2.0 (1.0 to 5.0) | 0.999 |
|  |  | Day 56 | 3.0 ± 1.3 3.0 (1.0 to 6.0) | 2.7 ± 1.3 2.0 (1.0 to 5.0) | 1.000 |
|  |  | Pre-Vaccination  (Baseline to Day 28) | 0.1 ± 1.2 0.0 (-2.0 to 2.0) 1.000 | 0.2 ± 1.0 0.0 (-2.0 to 2.0) 0.998 | 1.000 |
|  |  | Baseline to Day 56 | 0.3 ± 1.5 0.0 (-2.0 to 4.0) 0.839 | 0.5 ± 1.3 0.0 (-3.0 to 4.0) 0.487 | 1.000 |
|  |  | Post-Vaccination  (Day 28 to Day 56) | 0.2 ± 0.7 0.0 (-1.0 to 2.0) 0.988 | 0.3 ± 1.0 0.0 (-1.0 to 3.0) 0.922 | 1.000 |
| 29 | I feel no desire to do anything | Baseline | 2.1 ± 1.0 2.0 (1.0 to 4.0) | 1.8 ± 1.1 1.0 (1.0 to 4.0) | 0.994 |
|  |  | Day 28 | 2.1 ± 1.1 2.0 (1.0 to 4.0) | 1.9 ± 1.1 2.0 (1.0 to 4.0) | 1.000 |
|  |  | Day 56 | 2.3 ± 1.1 2.0 (1.0 to 5.0) | 2.3 ± 1.4 2.0 (1.0 to 6.0) | 1.000 |
|  |  | Pre-Vaccination  (Baseline to Day 28) | -0.0 ± 1.1 0.0 (-3.0 to 3.0) 1.000 | 0.2 ± 1.2 0.0 (-3.0 to 3.0) 1.000 | 1.000 |
|  |  | Baseline to Day 56 | 0.2 ± 1.3 0.0 (-2.0 to 3.0) 0.996 | 0.6 ± 1.3 0.0 (-3.0 to 4.0) 0.352 | 0.995 |
|  |  | Post-Vaccination  (Day 28 to Day 56) | 0.2 ± 1.0 0.0 (-2.0 to 3.0) 0.994 | 0.4 ± 1.1 0.0 (-1.0 to 4.0) 0.738 | 0.993 |
| 30 | When I am doing something, I can concentrate quite well | Baseline | 5.7 ± 0.9 6.0 (4.0 to 7.0) | 5.6 ± 1.1 6.0 (2.0 to 7.0) | 1.000 |
|  |  | Day 28 | 5.3 ± 1.2 5.0 (2.0 to 7.0) | 5.5 ± 1.3 5.5 (3.0 to 7.0) | 1.000 |
|  |  | Day 56 | 5.6 ± 0.7 6.0 (4.0 to 7.0) | 5.5 ± 1.2 6.0 (2.0 to 7.0) | 1.000 |
|  |  | Pre-Vaccination  (Baseline to Day 28) | -0.3 ± 1.3 0.0 (-4.0 to 1.0) 0.657 | -0.1 ± 1.0 0.0 (-2.0 to 2.0) 1.000 | 0.996 |
|  |  | Baseline to Day 56 | -0.0 ± 0.9 0.0 (-1.0 to 2.0) 1.000 | -0.0 ± 1.0 0.0 (-3.0 to 3.0) 1.000 | 1.000 |
|  |  | Post-Vaccination  (Day 28 to Day 56) | 0.3 ± 1.2 0.0 (-1.0 to 4.0) 0.882 | 0.1 ± 1.0 0.0 (-2.0 to 3.0) 1.000 | 0.996 |
